# Optimal Sliding Mode Fault-Tolerant Control for Multiple Robotic Manipulators via Critic-Only Dynamic Programming

**DOI:** 10.3390/s25175410

**Published:** 2025-09-02

**Authors:** Xiaoguang Zhang, Zhou Yang, Haitao Liu, Xin Huang

**Affiliations:** 1 School of Mechanical Engineering, Guangdong Ocean University, Zhanjiang 524088, China; 15521040206@stu.gdou.edu.cn (X.Z.); gdliuht@126.com (H.L.); 2112005004@stu.gdou.edu.cn (X.H.); 2 Guangdong Engineering Technology Research Center of Ocean Equipment and Manufacturing, Zhanjiang 524088, China; 3 Shenzhen Institute of Guangdong Ocean University, Shenzhen 518120, China

**Keywords:** multiple robotic manipulators, fault-tolerant control, quantitative prescribed performance control (QPPC), adaptive gain integral terminal sliding mode control (AGITSMC), critic-only neural network optimal dynamic programming (CNNODP)

## Abstract

This paper proposes optimal sliding mode fault-tolerant control for multiple robotic manipulators in the presence of external disturbances and actuator faults. First, a quantitative prescribed performance control (QPPC) strategy is constructed, which relaxes the constraints on initial conditions while strictly restricting the trajectory within a preset range. Second, based on QPPC, adaptive gain integral terminal sliding mode control (AGITSMC) is designed to enhance the anti-interference capability of robotic manipulators in complex environments. Third, a critic-only neural network optimal dynamic programming (CNNODP) strategy is proposed to learn the optimal value function and control policy. This strategy fits nonlinearities solely through critic networks and uses residuals and historical samples from reinforcement learning to drive neural network updates, achieving optimal control with lower computational costs. Finally, the boundedness and stability of the system are proven via the Lyapunov stability theorem. Compared with existing sliding mode control methods, the proposed method reduces the maximum position error by up to 25% and the peak control torque by up to 16.5%, effectively improving the dynamic response accuracy and energy efficiency of the system.

## 1. Introduction

With the continuous progress in intelligence, big data, cloud computing, and other technologies, the number of robotic manipulators will increase from a single function to multiple functions and from fixed scenes to flexible applications, whose applications in the fields of intelligent manufacturing, medical care, logistics, and other fields will continue to expand [1,2,3]. Therefore, multiple robotic manipulators will be more intelligent and have a complex direction of development. In the process of developing multiple robotic manipulators, fault-tolerant control is particularly important because of its complexity and interdependence among multiple robotic manipulators, which has also been one of the research focuses of cooperative control of multiple robotic manipulators in recent years [4,5,6].

Multiple robotic manipulators are critical for industrial and hazardous operations (e.g., nuclear waste handling [7,8]), but their control performance is limited by actuator/sensor failures, time-varying joint friction, and persistent disturbances [9]. High radiation in such environments directly causes sensor/actuator malfunctions, degrading system reliability. Therefore, enhancing the control performance of multiple robotic manipulators has become an urgent requirement. Sliding mode control (SMC) [10], known for its fast response and strong anti-interference capabilities, has emerged as a critical candidate for robotic manipulator system control [11,12]. However, existing SMC strategies face three fundamental contradictions in practical applications. First, there is a contradiction between robustness and precision. The traditional SMC is sensitive to parameter variations and external disturbances. For example, Wu et al.’s performance-constrained SMC method [13], when applied to complex terrain transportation tasks, experiences significant trajectory deviations due to inertial parameter fluctuations caused by load changes and wind disturbances. Although the boundary layer strategy [14,15] mitigates chattering, it compromises sliding mode stability, failing to meet the millimeter-level precision requirements for medical rehabilitation robotic manipulator end-effectors. Second, there is a conflict between computational complexity and real-time performance. Advanced SMC methods such as higher-order terminal SMC [16] rely on complex derivative calculations, significantly increasing the computational burden of the controller. In collaborative welding scenarios requiring a millisecond-level response, computational delays can lead to welding defects such as uneven seams or porosity [16,17]. Similarly, real-time performance degradation in multiple robotic manipulator collaborative grasping tasks causes asynchronous movements among manipulators. The third limitation is the limitations of adaptive capability in scenario adaptation. Existing adaptive SMC strategies perform well in specific scenarios but have deficiencies in robotic manipulator collaborative tasks: the terminal SMC in [18], which is effective for simple mobile robots, cannot adapt to dynamic parameter adjustments in industrial assembly; the nonsingular terminal strategy in [19] exhibits excessive complexity when handling strong coupling disturbances; and the stiffness-scheduling method in [20] lacks multiple robotic manipulator coordination mechanisms, hindering personalized treatment in rehabilitation scenarios. In summary, current SMC methods face significant limitations in robustness, real-time performance, and adaptive capability when applied to multiple robotic manipulators, as they fail to fully address their complex characteristics and domain-specific requirements. Therefore, developing a novel SMC structure with high robustness, low computational complexity, and strong scenario adaptability is crucial for advancing the stable application of multiple robotic manipulators in complex environments.

As the concept of sustainable development has taken deep root in people’s minds, the optimal control [21] of multiple robotic manipulators has become a key indicator for evaluating their performance. Reinforcement learning (RL), with its ability to obtain optimal strategies through continuous training, has garnered widespread attention in the field of multiple robotic manipulator control. To address the challenging problem of optimal control for multiple robotic manipulators under unknown nonlinear disturbances, reference [22,23,24] pioneered the adoption of an actor–critic architecture based on neural networks. In this architecture, the critic is responsible for calculating the cost function to evaluate control performance, while the actor continuously optimizes its own strategy on the basis of feedback from the critic, thereby gradually approaching the optimal solution. When the dynamic characteristics of the system are unknown, the identifier becomes a crucial component for accurately estimating uncertainties. References [25,26] introduced neural networks and fuzzy logic systems, respectively, as identifiers, effectively enhancing the system’s adaptability to unknown environments. However, the model uncertainties inherent in multiple robotic manipulator systems, along with the nonlinear characteristics of reinforcement learning, pose significant challenges in terms of heavy computational burdens for traditional optimal control methods in practical applications. To overcome this dilemma, refs. [27,28,29,30] innovatively proposed a critic-only reinforcement learning framework. Reference [27] ingeniously transformed the safety coordination problem into an optimal control problem by simplifying the structure of the critic neural network and introducing obstacle avoidance variables to increase system safety. Ma et al. [28] combined adaptive dynamic programming (ADP) algorithms with event-triggered mechanisms, utilizing a critic-only neural network to efficiently solve event-triggered Hamilton–Jacobi–Bellman equations, achieving decentralized tracking control. Reference [29] further constructed a critic-only neural network on the basis of policy iteration and ADP algorithms, successfully deriving an approximate fault-tolerant position—force optimal control strategy. Reference [30] targeted robotic manipulator systems with asymmetric input constraints and disturbances, achieving optimal control in complex environments by introducing a value function and approximately solving Hamilton–Jacobi–Isaacs equations online on the basis of ADP principles. Inspired by these studies, this paper focuses on multiple robotic manipulator systems in complex environments and designs an RL optimal control strategy based on a critic-only neural network. This strategy reduces computational complexity by optimizing the structure of the critic network and proposes explicit solutions to address the coupling issues of model uncertainties and nonlinear disturbances, providing a more efficient solution for practical deployment.

Prescribed performance control (PPC) [13,31] serves as an efficient control strategy capable of ensuring that system states adhere strictly to predefined performance specifications. Its core principle lies in the meticulous design of performance functions, combined with error transformation mechanisms and controller design [32,33], enabling control systems to precisely meet established performance requirements. Owing to this significant advantage, PPC has been extensively researched and applied in the field of robotic manipulator control. In terms of PPC implementation, reference [34,35,36] introduced performance functions to overcome performance limitations associated with tracking errors and transformed the error constraint problem of multiple robotic manipulators into an unconstrained stability control problem through error transformation strategies, providing new insights for system performance optimization. To address the state and output constraints of robotic manipulators, references [37,38] innovatively proposed barrier Lyapunov functions within the PPC framework, effectively ensuring that system states remain within predefined constraint boundaries. Furthermore, Liu et al. [39] developed a dynamic threshold finite-time prescribed performance control (DTFTPPC) method, which dynamically adjusts performance thresholds when the system reaches predefined time points, continuously compressing errors into smaller ranges and significantly enhancing control precision. However, existing research still presents limitations: the aforementioned multiple robotic manipulator systems require validation of initial position compliance with performance constraints before each operation, an additional verification step that increases operational complexity. Therefore, designing PPC control schemes that do not rely on initial position checks—while maintaining control performance and simplifying operational procedures—has become a critical challenge in the field of multiple robotic manipulator control, warranting in-depth and systematic investigations.

Inspired by the above discussion, an ADP based on an approximate optimal solution is proposed for the problem of actuator faults of multiple robotic manipulators in external disturbances. The sliding mode variable is constructed by combining the QPPC, and then the sliding mode variable is added to the value function to obtain an approximate optimal solution for the multiple robotic manipulators. The ADP is constructed to improve the control performance of multiple robotic manipulators under external disturbances and actuator failures. The contributions of this paper are summarized as follows.

(1) To address the problem of complex operations because of different initial states, a quantitative prescribed performance control (QPPC) strategy is introduced to release the initial state to realize flexible performance constraints and global prescribed performance.

(2) Adaptive gain-integrated terminal sliding mode control (AGITSMC) is proposed on the basis of a gain parameter that varies with the magnitude of the error, which improves the sensitivity of the sliding mode variable in complex environments. The proposed AGITSMC not only improves the convergence velocity and tracking accuracy but also enhances the stability under external disturbances and actuator faults.

(3) A critic-only neural network optimal dynamic programming (CNNODP) strategy is constructed by combining the gradient descent strategy, parallel learning technique, and experience playback technique. Unlike the traditional actor–critic or identifier–actor–critic NN structure, a critic-only NN is used to approximate the cost function, and the RL residuals and historical samples are employed to drive the update of the neural network, which achieves optimal control with fewer computations.

The remaining parts of this paper are structured as follows: Section 2 expounds on the dynamic model of multiple robotic manipulators and the theorems employed in this study; Section 3 provides a detailed derivation of the proposed control algorithms, namely, QPPC, AGITSMC, CNNODP, and fault compensation control, along with a stability analysis of the closed-loop system; Section 4 introduces the simulation setup and presents many simulation results to validate the effectiveness and robustness of the proposed algorithms; and finally, Section 5 summarizes the major findings of this paper and discusses potential future research directions.

## 2. Preliminaries

For ease of understanding, we use specific characters to represent the state variables of the robotic manipulator, with detailed explanations provided in the Table 1 below:

### 2.1. System Model

The dynamical models of multiple robotic manipulator systems are represented in the Euler–Lagrange form; here, we consider a multiple robotic manipulator system with N degrees of freedom with the following nonlinear differential equation:

(1) $$M_{i}(q_{i}){\ddot{q}}_{i}+C_{i}(q_{i},{\dot{q}}_{i}){\dot{q}}_{i}+G_{i}(q_{i})=\Gamma_{i}+\tau_{i}$$

**Remark 1.** 
*To further deepen the in-depth understanding of multiple robotic manipulator system models, a detailed and systematic explanation of the kinematic and dynamic models of multiple robotic manipulator systems is presented below.*

(1) Variables and models: The kinematic model links the joint space and operational space of the manipulator. For an N-degree-of-freedom manipulator, the relationship between the joint position vector $q_{i,s}$ and the end-effector pose is nonlinear because of the complex link–joint geometry. Forward kinematics calculates the end-effector pose from given joint positions via coordinate transformations, whereas inverse kinematics finds joint positions for a desired end-effector pose, which often has multiple or no solutions and requires specific algorithms. The dynamic model is key to understanding the forces on the manipulator. The vectors $q_{i,s}$, ${\dot{q}}_{i,s}$, and ${\ddot{q}}_{i,s}$ represent the position, velocity, and acceleration, respectively. The inertia matrix $M(q_{i})\in {ℜ}^{n\times n}$ connects acceleration to inertial forces and changes with joint position. The centripetal-C Coriolis matrix $C(q_{i},{\dot{q}}_{i})\in {ℜ}^{n\times n}$ accounts for velocity-related dynamic effects. The gravity vector $G(q_{i})\in {ℜ}^{n}$ shows that gravitational forces vary with pose. The input torque $\tau_{i}\in {ℜ}^{n}$ is the control signal, and the bounded disturbance vector $\Gamma_{i}\in {ℜ}^{n}$ represents the uncertainties. Newton’s second law shows that the left-hand side describes internal dynamics and that the right-hand side represents external forces.

(2) Assumptions and physical constraints: Links are assumed to be rigid to ease force calculations. Joints are ideal with no flexibility or clearance for direct motion transfer. Motion is assumed to be continuous without sudden impacts when smooth models are used. Dynamic parameters such as mass and inertia are considered known and constant, ignoring real-world variations. Nonlinear factors such as friction are often ignored initially, and the system is assumed to be linear or linearizable for applying linear control theory. The joint motion range is limited by mechanical design to prevent damage. Velocity is constrained by motor and structural performance, as high speed can cause vibration and stress. The input torque is limited by the motor capacity to avoid overloading. Acceleration is restricted by system inertia and motor capabilities. Manipulators must avoid collisions to prevent damage and follow physical laws such as energy conservation.

(3) Reasons for ignoring parameter uncertainty: Multiple robotic manipulator models are complex, and adding parameter uncertainty makes them even more complex, increasing the difficulty of analysis and controller design. In ideal scenarios such as well-controlled labs, accurate parameter knowledge is reasonable for focusing on basic system aspects. A phased research approach is common: first, a basic controller is designed without considering uncertainty for fundamental functions; then, robustness against uncertainty is enhanced later for an efficient research process.

Combined with the dynamic Equation (1) of multiple robotic manipulators, one obtains

(2) $${\ddot{q}}_{i}=M_{i}^{-1}(q_{i})\left(\tau_{i}+\Gamma_{i}-C_{i}(q_{i},{\dot{q}}_{i}){\dot{q}}_{i}-G_{i}(q_{i})\right)$$

For simplicity, we let $H_{i}=M_{i}^{-1}(q_{i})$ and $D_{i}=\left(\Gamma_{i}-C_{i}(q_{i},{\dot{q}}_{i}){\dot{q}}_{i}-G_{i}(q_{i})\right)$. The expression for ${\ddot{q}}_{i}$ can be rewritten as ${\ddot{q}}_{i}=H_{i}D_{i}+H_{i}\tau_{i}$. Let $H_{ti}$ denote the diagonal matrix of matrix $H_{i}$; we obtain $H_{i}=H_{ti}+H_{Ti}$, where ${\ddot{q}}_{i}=H_{ti}\tau_{i}+H_{Ti}\tau_{i}+H_{i}D_{i}$. Thus, ${\ddot{q}}_{i}$ can again be rewritten as follows:

(3) $${\ddot{q}}_{i}=H_{ti}\tau_{i}+{\mathbb{Q}}_{i}$$

where ${\mathbb{Q}}_{i}=H_{Ti}\tau_{i}+H_{i}D_{i}$.

The following actuator fault model is constructed:

(4) $$\tau_{i,s}=\Phi_{i,s}^{T}\tau_{hi,s}+{℘}_{i,s}$$

where $\tau_{hi,s}$ and $\tau_{i,s}$ denote the input and output of the fault model, respectively; $\Phi_{i,s}$ and ${℘}_{i,s}$ denote the bias fault and additive fault of the actuator, respectively; and $\Phi_{i,s}\in (0,1]$, where ${*}_{i}={\left[{*}_{i1},{*}_{i2},\ldots ,{*}_{iN}\right]}^{T}$ and ${*}_{i}$ denote the state vectors of the i−th robotic manipulator, with a total of n dimensions. s=1,2,…,N, denotes the degree of freedom of the i−th robotic manipulator with N dimensions.

### 2.2. Graph Description

Define the followers set by $f=\left\{1,\ldots ,N\right\}$. The digraph $\chi =\left(K,E,A\right)$ is used to represent the relation under which information about the state is exchanged among robotic manipulators, where $A=\left[a_{ij}\right]\in {ℜ}^{n\times n}$ represents the communication of followers. $a_{ij}=1$ indicates that the i robotic manipulator can obtain information from the j robotic manipulator; otherwise, $a_{ij}=0$. The set of followers is defined as $K=\left\{k_{1},k_{2},\ldots ,k_{n}\right\}$. $d_{i}=\sum_{j=1}^{n}a_{ij}$ and D = diag{$d_{i}$}  i=1,…,n are defined. The Laplacian matrix is defined as $L^{\prime }=D-A$. The adjacency matrix of leaders is defined as B = diag{$b_{i}$}, which represents the communication among the leader and the followers. $b_{i}=1$ indicates that the i robotic manipulator can obtain information from the leader; otherwise, $b_{i}=0$. For later analysis, the information exchange matrix is defined as $H=L^{\prime }+B$.

### 2.3. Radial Basis Function Neural Network (RBFNN) Research

In current research on control systems, uncertain nonlinear terms in nonlinear systems can be approximated via the RBFNN.

In general, the RBFNN is utilized to approximate any unknown function F(ξ). The RBFNN is denoted as follows:

(5) $$F(\xi )=W^{*T}S(\xi )+\chi (\xi )$$

where $\xi ={\left[\xi_{1},\xi_{2},\ldots ,\xi_{n}\right]}^{T}\in {ℜ}^{n}$ and $W={\left[W_{1},W_{2},\ldots W_{n}\right]}^{T}\in {ℜ}^{n}$ indicate the unknown input vector and the weight vector, respectively. $S(\xi )={\left[S_{1}(\xi ),S_{2}(\xi ),\ldots S_{n}(\xi )\right]}^{T}\in {ℜ}^{n}$ is the known basis function vector. F(ξ) is a continuous function that is defined on a compact set Λ. Hence, for $\forall \bar{\chi }>0$, a neural network exists such that $\left|F(\xi )-W^{T}S(\xi )\right|\leq \bar{\chi }$. Therefore, $\left|\chi (\xi )\right|\leq \bar{\chi }$. The optimal constant vector $W^{*}$ is designed to be $W^{*}=\arg_{\underset{W\in {ℜ}^{M}}{\min}}\left\{\sup\left|F(\xi )-W^{T}S(\xi )\right|\right\}$.

### 2.4. Lemmas and Assumptions

**Lemma 1** 
([40])**.** *For any constants $\Theta_{i,1}>0$ and $\Theta_{i,2}>0$, the relationships below can be obtained:*

(6) $$0<\Theta_{i,2}I_{i,n}<M_{i}<\Theta_{i,1}I_{i,n}$$

**Lemma 2** 
([41])**.** *For any continuous functions x(t) and $x_{0}(t)$ on [0,∞) that satisfy $\underset{t\to \infty }{\lim}x_{0}(t)=0$ and $x_{0}(t)>0$, the relationships below can be obtained:*

(7) $$\left|x(t)\right|<x_{0}(t)+\frac{x^{2}(t)}{\sqrt{x^{2}(t)+x_{0}^{2}(t)}}$$

**Assumption 1.**  *The bias $\Phi_{i,s}$ fault and additive ${℘}_{i,s}$ fault of the actuator are bounded, i.e., the existence of constants $r_{1}$ and $r_{2}$ such that $\Phi_{i,s}\leq r_{1}$ and $\left|{℘}_{i,s}\right|\leq r_{2}$*.

**Assumption 2**  ([27])**.** *The activation function $\varphi_{i,s}$ and its gradient $\nabla \varphi_{i,s}$ are paradigmatically bounded, i.e., $\varphi_{i,s}\leq {\bar{\varphi }}_{i,s}$ and $\nabla \varphi_{i,s}\leq \nabla {\bar{\varphi }}_{i,s}$, where ${\bar{\varphi }}_{i,s}$ and $\nabla {\bar{\varphi }}_{i,s}$ are unknown positive parameters*.

## 3. Main Results

**Figure 1:**
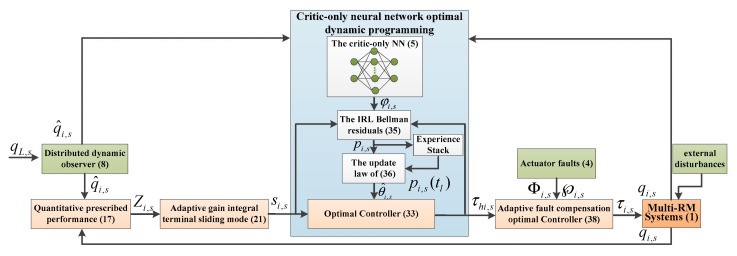
Overall control scheme of the proposed method.

The main objective of this work is to satisfy the control performance requirements of multiple robotic manipulators with actuator faults and external disturbances. The overall control scheme of this paper is shown in Figure 1. First, a quantitative prescribed performance control (QPPC) strategy is designed by combining the signals generated by the observer. Then, the QPPC is utilized to construct the adaptive gain-integrated terminal sliding mode control (AGITSMC). Third, the sliding mode variables are introduced into the value function to construct a critic-only neural network optimal dynamic programming (CNNODP) control strategy for approximating the optimal solution. Finally, adaptive fault compensation control is proposed to reconstruct controllers with fault problems.

### 3.1. Quantitative Prescribed Performance Control (QPPC)

In general, the robotic manipulator connected to the leader knows its information, whereas other robotic manipulators do not have direct access to the leader’s information. However, once the robotic manipulator connected to the leader is faulty, the other robotic manipulators are inevitably greatly affected. To ensure that all the robotic manipulators can directly access the leader information without being affected by other robotic manipulators, the dynamic distribution observer is designed as follows:

(8) $${\dot{\hat{q}}}_{i,s}=v_{i,s}-g_{1}\sum_{j\in N}a_{i,j}({\hat{q}}_{i,s}-{\hat{q}}_{j,s})-g_{1}b_{i}({\hat{q}}_{i,s}-q_{L,s})$$

(9) $${\dot{v}}_{i,s}=h_{i,s}-g_{2}\sum_{j\in N}a_{i,j}({\hat{q}}_{i,s}-{\hat{q}}_{j,s})-g_{2}b_{i}({\hat{q}}_{i,s}-q_{L,s})-g_{3}\sum_{j\in N}a_{i,j}(v_{i,s}-v_{j,s})-g_{3}b_{i}(v_{i,s}-v_{L,s})$$

(10) $${\dot{h}}_{i,s}=-\frac{g_{4}}{b_{i}+\sum_{j\in N}a_{i,j}}(\sum_{j\in N}a_{i,j}(h_{i,s}-h_{j,s})+b_{i}(h_{i,s}-h_{0,s}))+(\frac{1}{b_{i}+\sum_{j\in N}a_{i,j}})(\sum_{j\in N}{\dot{h}}_{j,s}+b_{j}{\dot{h}}_{0,s})$$

where $q_{L,s}$, $v_{0,s}$, and $h_{0,s}$ denote the leader’s trajectory, velocity and acceleration, respectively; ${\hat{q}}_{i,s}$, $v_{i,s}$, and $h_{i,s}$ denote the follower’s trajectory, velocity and acceleration, respectively; and $g_{c}$ (c=1, 2, 3, 4) denotes positive parameters. The observation errors of the observers are defined as $E_{pi,s}={\hat{q}}_{i,s}-q_{L,s}$, $E_{vi,s}=v_{i,s}-v_{0,s}$, and $E_{hi,s}=h_{i,s}-h_{0,s}$.

**Lemma 3** 
([27])**.** *For ${\hat{q}}_{i,s}(0)$, $v_{i,s}(0)$ and $h_{i,s}(0)$ with any initial state satisfying $\left[\begin{array}{l} g_{1}H\;-I_{N}\\ g_{2}H\;g_{3}H \end{array}\right]>0$ and $g_{4}>0$, the observer’s state converges exponentially to the leader’s trajectory, i.e., ${\hat{q}}_{i,s}\to q_{L,s}$, $v_{i,s}\to v_{0,s}$ and $h_{i,s}\to h_{0,s}$ as t→∞*.

The angle error is given by the following:

(11) $$E_{i,s}=q_{i,s}-{\hat{q}}_{i,s}$$

The velocity error is given by the following:

(12) $$e_{i,s}={\dot{q}}_{i,s}-v_{i,s}$$

Here, by using the inverse tangent function, the error is quantified as follows:

(13) $$E_{ti,s}=\frac{2}{\pi }\arctan(E_{i,s})$$

On the basis of the inverse tangent function, $-1<\frac{2}{\pi }\arctan(E_{i,s})<1$.

To ensure the prescribed performance of the consensus error with multiple robotic manipulator positions, the prescribed performance function is designed as follows:

(14) $$\beta_{i,s}(t)=\left\{\begin{array}{l} \left(\beta_{oi,s}-\beta_{\infty i,s}){\left(\frac{T_{a}-t}{T_{a}}\right)}^{2}+\beta_{\infty i,s},t\in [0,T_{a}\right)\\ \beta_{\infty i,s},\;t\in [T_{a},\infty ) \end{array}\right.$$

where $\beta_{oi,s}>0$ and $\beta_{\infty i,s}>0$ denote the initial state and the ultimate convergence domain, respectively, and where $T_{a}$ is a predefined convergence time.

Deriving from $\beta_{i,s}(t)$, one can obtain the following:

(15) $${\dot{\beta }}_{i,s}(t)=\left\{\begin{array}{l} \frac{-2}{T_{a}}(\beta_{oi,s}-\beta_{\infty i,s})(\frac{T_{a}-t}{T_{a}}),t\in [0,T_{a})\\ 0,\;t\in [T_{a},\infty ) \end{array}\right.$$

To ensure PPC, by combining the prescribed performance Function (14) and quantified Function (13), the error transformation function is defined as follows:

(16) $$Z_{i,s}=\tan(\frac{\pi E_{ti,s}}{2\beta_{i,s}})=\tan(\frac{\arctan(E_{i,s})}{\beta_{i,s}})$$

**Remark 2.** 
*The error transformation function $\tan(\frac{\pi }{2}x)$ ensures that −1<x<1 for −1<x(0)<1. $\beta_{i,s}(t)$ is a prescribed performance function of $\arctan(E_{i,s})$ and $-\frac{\pi }{2}\beta_{i,s}(0)<\arctan(E_{i,s}(0))<\frac{\pi }{2}\beta_{i,s}(0)$. From the above, it follows that $\beta_{i,s}(t)$ is a monotonically decreasing function.$-\frac{\pi }{2}\beta_{i,s}(t)<\arctan(E_{i,s}(t))<\frac{\pi }{2}\beta_{i,s}(t)$ always holds under the constraints of $\tan(\frac{\pi }{2}x)$, and $\arctan(E_{i,s})$ enters and is always in the predefined domain $\left[-\beta_{\infty i,s},\beta_{\infty i,s}\right]$. On the basis of the properties of the inverse tangent function, $\arctan(E_{i,s})$ always satisfies $-1<\frac{2}{\pi }\arctan(E_{i,s})<1$. Thus, $E_{i,s}(t)$ is unconstrained when $\beta_{i,s}(t)\geq 1$, and $E_{i,s}(t)$ is constrained as $\beta_{i,s}(t)$ decreases and satisfies $\beta_{i,s}(t)<1$*.

From the above, the constraint states of $E_{i,s}(t)$ can be classified into two types.

1. When the prescribed performance function $\beta_{i,s}(t)\geq 1$, one can obtain the following:
  (17) $$-\infty <E_{i,s}<\infty$$
2. When the prescribed performance function $\beta_{i,s}(t)<1$, one can obtain the following:
  (18) $$-\tan(\frac{\pi }{2}\beta_{i,s})<E_{i,s}<\tan(\frac{\pi }{2}\beta_{i,s})$$

Therefore, the PPC strategy designed in this paper releases the initial condition of multiple robotic manipulators, which no longer needs to know the initial state.

Deriving from $Z_{i,s}$, one can obtain the following:

(19) $${\dot{Z}}_{i,s}=r_{i,s}{\dot{E}}_{i,s}-R_{i,s}$$

where $r_{i,s}=\sec^{2}(\frac{\arctan(E_{i,s})}{\beta_{i,s}})\frac{1}{\beta_{i,s}(1+E_{i,s}^{2})}>0$ and $R_{i,s}=\sec^{2}(\frac{\arctan(E_{i,s})}{\beta_{i,s}})\frac{\arctan(E_{i,s}){\dot{ℵ}}_{i,s}}{\beta_{i,s}^{2}}$.

**Remark 3.** 
*Combined with the quantization function, a new error transformation function is developed in this paper. Unlike the traditional error transformation strategy* [34,35,36,37,38,39]*, this strategy releases the initial condition and no longer requires the initial error to be within a certain domain, realizing global PPC. In addition, with the help of the quantization function, the error is transformed to a smaller bounded domain even if the error is large, which can greatly reduce the control energy and overshooting phenomenon.*

### 3.2. Adaptive Gain Integral Terminal Sliding Mode Control (AGITSMC)

To ensure that the system trajectory converges quickly and with good robustness, an AGITSMC control strategy is designed as follows:

(20) $$\sigma_{i,s}=b_{1i,s}Z_{i,s}+b_{2i,s}{\dot{Z}}_{i,s}+b_{3}\int_{0}^{t}\left(b_{1i,s}Z_{i,s}+b_{2i,s}{\dot{Z}}_{i,s}\right)dr$$

where $b_{1i,s}=\frac{a_{1}}{{\left(2-{\left(\frac{2}{\pi }\arctan(E_{i,s})\right)}^{2}\right)}^{2c_{1}}}>0$ and $b_{2i,s}=\frac{a_{2}}{{\left(2-{\left(\frac{2}{\pi }\arctan(e_{i,s})\right)}^{2}\right)}^{2c_{2}}}>0$, and where $a_{1}$, $a_{2}$, $b_{3}$, $c_{1}$, and $c_{2}$ are design parameters.

Thus, the derivative of $\sigma_{i,s}$ is as follows:

(21) $$\begin{array}{l} {\dot{\sigma }}_{i,s}=b_{1i,s}{\dot{Z}}_{i,s}+{\dot{b}}_{1i,s}Z_{i,s}+b_{2i,s}{\ddot{Z}}_{i,s}+{\dot{b}}_{2i,s}{\dot{Z}}_{i,s}+b_{3}(b_{1i,s}Z_{i,s}+b_{2i,s}{\dot{Z}}_{i,s})\\ \;=F_{i,s}\tau_{i,s}+f_{i,s} \end{array}$$

where $f_{i,s}=b_{2i,s}({\mathbb{Q}}_{i,s}r_{i,s}+{\dot{r}}_{i,s}{\dot{E}}_{i,s}-R_{i,s}-u_{i,s}{\ddot{\hat{q}}}_{i,s})+b_{1i,s}{\dot{Z}}_{i,s}+{\dot{b}}_{1i,s}Z_{i,s}+{\dot{b}}_{2i,s}{\dot{Z}}_{i,s}+b_{3i,s}((b_{1i,s}Z_{i,s}+b_{2i,s}{\dot{Z}}_{i,s}))$ and $F_{i,s}=b_{2i,s}r_{i,s}H_{ti,s}$.

On the basis of the [42] SMC principle, when the system runs into the sliding stage, i.e., $\sigma_{i,s}=0$, the following can be obtained:

(22) $$b_{1i,s}Z_{i,s}+b_{2i,s}{\dot{Z}}_{i,s}+b_{3}\int_{0}^{t}\left(b_{1i,s}Z_{i,s}+b_{2i,s}{\dot{Z}}_{i,s}\right)dr=0$$

**Remark 4.**  *The SMC process is considered in two stages* [42]*: the reaching stage and the sliding mode control stage. However, the trajectory tends to depart from the slide modeling surface under the effects of other adverse conditions, such as obstacle avoidance, external disturbances, actuator faults, and input saturation, which leads to the problem of robustness degradation of the system, especially in the reaching phase. Obviously, the problem of robustness degradation severely affects the control performance. In this paper, AGITSMC control is developed to suppress the jitter of the system. On this basis, the adaptive gain parameters $b_{1i,s}$ and $b_{2i,s}$ are introduced, which increase when the error increases and decrease when the error decreases. Therefore, the introduction of the adaptive gain parameter avoids excessive or insufficient gain, which not only improves the convergence velocity and control accuracy but also reduces the jitter phenomenon. Thus, the proposed AGITSMC strategy can enable the system to maintain good control performance in complex environments.*

### 3.3. Critic-Only Neural Network Optimal Dynamic Programming (CNNODP) Control

Next, to design an optimal sliding mode controller, we construct a critic-only neural network (NN) strategy with the RBFNN, where the critic-only NN is updated in real time via RL Bellman residuals and empirical replay techniques. Combining the sliding variables in (20) with the total cost of the control input energy, the performance function index with an attenuation coefficient can be designed as follows:

(23) $$C_{i,s}(\sigma_{i,s}(t))=\int_{t}^{\infty }e^{-w(r-t)}N_{i,s}(\sigma_{i,s}(r),\tau_{i,s})dr$$

where $N_{i,s}(\sigma_{i,s},\tau_{i,s})=\sigma_{i,s}^{2}+\tau_{i,s}^{2}$ denotes the cost function and where $\tau_{i,s}$ denotes the controller. The attenuation coefficient w>0 and the $e^{-w(r-t)}$ term ensure that the cost function remains bounded even though the tracking errors do not eventually converge to 0.

**Remark 5.** 
*The purpose of adaptive optimal control is to minimize the value function of a particular characteristic. In nominal systems* [22,23,24,25,26]*, the cost function is a quadratic function related to the tracking error and control input. In this work, sliding mode variables are added to the value function of each robotic manipulator to achieve optimal sliding mode control to address the effects of environmental disturbances and actuator faults on control performance.*

To address the optimal trajectory problem, a new adaptive optimal sliding mode controller is designed. The design process is as follows:

First, the Bellman equation is obtained by using Leibniz’s law to derive the following Equation (23):

(24) $$\nabla C_{i,s}(\sigma_{i,s}){\dot{\sigma }}_{i,s}-wC_{i,s}(\sigma_{i,s})+N_{i,s}(\sigma_{i,s},\tau_{i,s})=0$$

where $\nabla C_{i,s}(\sigma_{i,s})=\frac{\partial C_{i,s}(\sigma_{i,s})}{\partial \sigma_{i,s}}$ is the gradient of $C_{i,s}(\sigma_{i,s})$ in the direction of $\sigma_{i,s}$.

Then, the Hamiltonian function can be defined as follows:

(25) $$H_{i,s}(\sigma_{i,s},\nabla C_{i,s}(\sigma_{i,s}),\tau_{i,s})=\nabla C_{i,s}(\sigma_{i,s})(F_{i,s}\tau_{i,s}+f_{i,s})-wC_{i,s}(\sigma_{i,s})+N_{i,s}(\sigma_{i,s},\tau_{i,s})$$

Ω(A) is defined as the set of permissible controls on A, where $A\in R^{n}$ denotes the tight set. The optimized cost function is as follows:

(26) $$C_{i,s}^{*}(\sigma_{i,s})=\underset{\tau \in \Omega (A)}{\min}\int_{t}^{\infty }e^{-w(r-t)}N_{i,s}(\sigma_{i,s}(r),\tau_{i,s})dr=\int_{t}^{\infty }N_{i,s}(\sigma_{i,s}(r),\tau_{i,s}^{*})dr$$

Thus, the Hamilton–Jacobi–Bellman (HJB) equation can be designed according to the following:

(27) $$H_{i,s}(\sigma_{i,s},\nabla C_{i,s}^{*}(\sigma_{i,s}),\tau_{i,s}^{*})=\nabla C_{i,s}^{*}(\sigma_{i,s})(F_{i,s}\tau_{i,s}^{*}+f_{i,s})-wC_{i,s}^{*}(\sigma_{i,s})+N_{i,s}(\sigma_{i,s},\tau_{i,s}^{*})$$

where $\nabla C_{i,s}^{*}(\sigma_{i,s})=\frac{\partial C_{i,s}^{*}(\sigma_{i,s})}{\partial \sigma_{i,s}}$ is the gradient of $C_{i,s}^{*}(\sigma_{i,s})$ in the direction of $\sigma_{i,s}$.

${\ddot{q}}_{i,s}$ is considered optimized $\tau_{i,s}^{*}$. By solving for $\frac{\partial H_{i,s}}{\partial \tau_{i,s}^{*}}=0$, the optimal controller can be calculated as follows:

(28) $$\tau_{i,s}^{*}=-\frac{F_{i,s}}{2}\nabla C_{i,s}^{*}(\sigma_{i,s})$$

Obviously, the direct computation of the optimal controller is difficult because of the complex nonlinearities in $\nabla C_{i,s}^{*}(\sigma_{i,s})$. To achieve the optimal performance control objective, the term $C_{i,s}^{*}(\sigma_{i,s})$ is constructed as $C_{i,s}^{*}(\sigma_{i,s})=\lambda_{i,s}\vartheta_{i,s}^{2}+C_{i,s}^{0}(\sigma_{i,s})$, where $\lambda_{i,s}$ a is a positive number.

Therefore, one can obtain the following:

(29) $$\nabla C_{i,s}^{*}(\sigma_{i,s})=2\lambda_{i,s}\sigma_{i,s}+\nabla C_{i,s}^{0}(\sigma_{i,s})$$

Therefore, $\tau_{i,s}^{*}$ can be rewritten as follows:

(30) $$\tau_{i,s}^{*}=-\lambda_{i,s}F_{i,s}\sigma_{i,s}-\frac{1}{2}F_{i,s}\nabla C_{i,s}^{0}(\sigma_{i,s})$$

Considering the unknown functions, the critic-only NN is used to approximate $C_{i,s}^{0}(\sigma_{i,s})$ and $\nabla C_{i,s}^{0}(\sigma_{i,s})$. The cost function is approximated as $C_{i,s}^{0}(\vartheta_{i,s})=\theta_{i,s}^{T}\varphi_{i,s}+\xi_{i,s}$ and $\nabla C_{i,s}^{0}(\sigma_{i,s})=\varphi_{i,s}^{T}\theta_{i,s}+\xi_{i,s}$, where $\theta_{i,s}$ is the ideal NN parameter vector, $\varphi_{i,s}$ is the activation function, and $\xi_{i,s}$ denotes the approximation error with $\xi_{i,s}\leq {\bar{\xi }}_{i,s}$. Since $\theta_{i,s}$ is unknown, the critic-only NN is calculated as follows:

(31) $$\nabla {\hat{C}}_{i,s}^{0}(\sigma_{i,s})=\nabla \varphi_{i,s}^{T}{\hat{\theta }}_{i,s}$$

where $\nabla {\hat{C}}_{i,s}^{0}(\sigma_{i,s})$ and ${\hat{\theta }}_{i,s}$ denote the cost function and the critic-only NN weight $\theta_{i,s}$ estimate, respectively. ${\tilde{\theta }}_{i,s}={\hat{\theta }}_{i,s}-\theta_{i,s}$ and ${\tilde{\theta }}_{i,s}$ are the estimate errors.

Finally, combining (30) and (31), the approximate optimal controller can be rewritten as follows:

(32) $$\tau_{i,s}=-\lambda_{i,s}F_{i,s}\sigma_{i,s}-\frac{1}{2}F_{i,s}\nabla \varphi_{i,s}^{T}{\hat{\theta }}_{i,s}$$

To update the critic-only NN in real time with the RL Bellman residuals and empirical replay techniques, we can define $t_{1}=t-T_{w}$ and $T_{w}>0$. By using the RL algorithm, Equation (23) can be rewritten as follows:

(33) $$C_{i,s}^{*}(\sigma_{i,s}(t_{1}))=\int_{t_{1}}^{t}e^{-w(r-t_{1})}N_{i,s}(\sigma_{i,s}(r),\tau_{i,s}^{*})dr+e^{-wT_{w}}C_{i,s}^{*}(\sigma_{i,s})$$

Therefore, the RL Bellman residuals caused by the critic-only NN can be defined as follows:

(34) $$\begin{array}{l} p_{i,s}=\int_{t_{1}}^{t}e^{-w(r-t_{1})}N_{i,s}(\sigma_{i,s}(r),\tau_{i,s})dr+e^{-wT_{w}}{\hat{C}}_{i,s}(\sigma_{i,s})-{\hat{C}}_{i,s}(\sigma_{i,s}(t_{1}))\\ \;=\int_{t_{1}}^{t}e^{-w(r-t_{1})}N_{i,s}(\sigma_{i,s}(r),\tau_{i,s})dr+e^{-wT_{w}}(\lambda_{i,s}\sigma_{i,s}^{2}+{\hat{\theta }}_{i,s}^{T}\varphi_{i,s})-(\lambda_{i,s}\sigma_{i,s}^{2}(t_{1})+{\hat{\theta }}_{i,s}^{T}\varphi_{i,s}(t_{1}))\\ \;=\int_{t_{1}}^{t}e^{-w(r-t_{1})}N_{i,s}(\sigma_{i,s}(r),\tau_{i,s})dr+\Delta \sigma_{i,s}+{\hat{\theta }}_{i,s}^{T}\Delta \varphi_{i,s} \end{array}$$

where $\Delta \varphi_{i,s}=e^{-wT_{w}}\varphi_{i,s}-\varphi_{i,s}(t_{1})$ and $\Delta \sigma_{i,s}=e^{-wT_{w}}\lambda_{i,s}\sigma_{i,s}^{2}-\lambda_{i,s}\sigma_{i,s}^{2}(t_{1})$.

**Remark 6.** 
*In this paper, the critic-only NN is constructed to obtain an approximate optimal controller instead of the actor–critic structure or the identifier–actor–critic structure. The proposed strategy is excellent over several existing strategies.*

First, in controller (32), the consensus control relies on the value function, and we use the critic-only NN to obtain the approximate optimal controller. Compared with the actor–critic structure [22,23,24], the critic-only NN structure adopted in this paper greatly reduces the control system complexity, which is favorable for practical application in engineering.

Next, compared with traditional adaptive optimal control [25,26], the RL Bellman residuals are adopted to drive the update law of the critic-only NN. However, because of the uncertainty of the system model, there are often unknown functions in the HJB equations, which requires the identifier network to estimate the unknown function ${\mathbb{Q}}_{i,s}(t)$ of (3). There is no doubt that this greatly increases the complexity of the system. To eliminate the identifier network, we use an empirical playback technique to obtain the RL residuals (34) and then drive the updating law with the help of the RL residuals. As a result, there is no need to consider the effect of unknown functions, which reduces the need for an identifier network and greatly reduces the complexity of the system.

Third, unlike [27,28,29,30], the designed update law contains two items. The first one is driven by using RL residuals on the basis of the gradient descent method. The second uses historical samples to adjust the weight vector, which accelerates the decrease in the adaptive law [43].

Finally, compared with existing reinforcement learning algorithms [22,23,24,25,26,27,28,29,30], this paper introduces a feedback term $-\lambda_{i,s}\sigma_{i,s}$ to improve the convergence velocity of robotic manipulators.

To minimize the RL residual, the gradient descent method, parallel learning technique, and experience replay technique are utilized to obtain the update law of ${\hat{\theta }}_{i,s}$ as follows:

(35) $${\dot{\hat{\theta }}}_{i,s}=-p_{0,s}\frac{\Delta \varphi_{i,s}}{{\left(1+\Delta \varphi_{i,s}^{T}\Delta \varphi_{i,s}\right)}^{2}}p_{i,s}-p_{0,s}\sum_{l=1}^{L}\frac{\Delta \varphi_{i,s}^{l}}{{\left(1+\Delta \varphi_{i,s}^{lT}\Delta \varphi_{i,s}^{l}\right)}^{2}}p_{i,s}^{l}$$

where $p_{0,s}$ is the learning rate, whose value determines the training velocity of the critic-only NN, and where $t_{l}\in \{t_{1},t_{2},\ldots ,t_{L}\}$ is the index that is used to mark the historical state of the storage.

According to ${\tilde{\theta }}_{i,s}={\hat{\theta }}_{i,s}-\theta_{i,s}$, one can obtain the following:

(36) $$\begin{array}{l} {\dot{\tilde{\theta }}}_{i,s}={\dot{\hat{\theta }}}_{i,s}=-p_{0,s}\frac{\Delta \varphi_{i,s}^{T}\Delta \varphi_{i,s}}{{\left(1+\Delta \varphi_{i,s}^{T}\Delta \varphi_{i,s}\right)}^{2}}{\tilde{\theta }}_{i,s}-p_{0,s}\sum_{l=1}^{L}\frac{\Delta \varphi_{i,s}^{lT}\Delta \varphi_{i,s}^{l}}{{\left(1+\Delta \varphi_{i,s}^{lT}\Delta \varphi_{i,s}^{l}\right)}^{2}}{\tilde{\theta }}_{i,s}\\ \;+p_{0,s}\frac{\Delta \varphi_{i,s}}{{\left(1+\Delta \varphi_{i,s}^{T}\Delta \varphi_{i,s}\right)}^{2}}\Delta \xi_{i,s}+p_{0,s}\sum_{l=1}^{L}\frac{\Delta \varphi_{i,s}^{l}}{{\left(1+\Delta \varphi_{i,s}^{lT}\Delta \varphi_{i,s}^{l}\right)}^{2}}\Delta \xi_{i,s}^{l} \end{array}$$

where $\Delta \xi_{i,s}=\xi_{i,s}(t_{1})-e^{-wT_{w}}\xi_{i,s}$ and $\Delta \xi_{i,s}^{l}=\xi_{i,s}^{l}(t_{1})-e^{-wT_{w}}\xi_{i,s}^{l}$.

**Remark 7.** 
*To ensure that ${\hat{\theta }}_{i,s}$ converges to $\theta_{i,s}$, this paper combines the gradient descent method and parallel learning technique to relax the PE condition. Therefore, the update law (35) is classified into two terms. The first is driven by the current data via the gradient descent algorithm, and the second is driven by the historical data via the gradient descent algorithm. To minimize the residuals from the RL, the current data and historical data are used together to drive the update of (35) via the parallel learning technique. Define ${\bar{\varphi }}_{i,s}^{l}=\frac{\Delta \varphi_{i,s}^{l}}{{\left(1+\Delta \varphi_{i,s}^{lT}\Delta \varphi_{i,s}^{l}\right)}^{2}}$ and $Z({\bar{\varphi }}_{i,s})={\left[{\bar{\varphi }}_{i,s}^{1},{\bar{\varphi }}_{i,s}^{2},\ldots ,{\bar{\varphi }}_{i,s}^{l}\right]}^{T}$ as the state of the history. To increase the velocity of convergence of the critic-only neural network and relieve the stringent incentive persistence (PE) conditions encountered in several existing studies, the number of stored history states can be defined as $L>rank(Z({\bar{\varphi }}_{i,s})))$, such as [43]*.

### 3.4. Adaptive Fault Compensation Control

Adaptive fault compensation control is designed here to address the problem of multiple robotic manipulator actuator faults. To achieve the control objective, an adaptive fault compensation optimal controller is designed as follows.

Combining (32), the compensation controller is calculated as follows:

(37) $$\tau_{hi,s}=-\lambda_{i,s}F_{i,s}\sigma_{i,s}-\frac{1}{2}F_{i,s}\nabla \varphi_{i,s}^{T}{\hat{\theta }}_{i,s}$$

(38) $$\alpha_{i,s}=\tau_{hi,s}-\frac{\sigma_{i,s}}{\sqrt{\sigma_{i,s}^{2}+k_{2,2}^{2}e^{-2t}}}{\hat{\gamma }}_{2i,s}$$

(39) $$\tau_{\gamma i,s}=({\hat{\gamma }}_{1i,s}+1)\alpha_{i,s}$$

Combining (37)–(39), the adaptive fault compensation optimal controller is calculated as follows:

(40) $$\tau_{i,s}=\Phi_{i,s}(\tau_{\gamma i,s})+{℘}_{i,s}$$

The adaptive parameters ${\hat{\gamma }}_{1}$ and ${\hat{\gamma }}_{2}$ are approximations of $\frac{1}{\gamma_{1}}$ and $\gamma_{2}$, respectively, which can be calculated as follows:

(41) $${\dot{\hat{\gamma }}}_{1i,s}=-k_{1,1}{\hat{\gamma }}_{1i,s}-k_{1,2}F_{i,s}\sigma_{i,s}\alpha_{i,s}$$

(42) $${\dot{\hat{\gamma }}}_{2i,s}=-k_{2,1}{\hat{\gamma }}_{2i,s}+\frac{\sigma_{i,s}^{2}F_{i,s}}{\sqrt{\sigma_{i,s}^{2}+k_{2,2}^{2}e^{-2t}}}$$

where $k_{1,1}$, $k_{1,2}$, $k_{2,1}$, and $k_{2,2}$ are positive constants; if ${\hat{\gamma }}_{1i,s}(0)>0$ and ${\hat{\gamma }}_{2i,s}(0)>0$, it is simple to obtain ${\hat{\gamma }}_{1i,s}(t)>0$ and ${\hat{\gamma }}_{2i,s}(t)>0$; ${\tilde{\gamma }}_{1}={\hat{\gamma }}_{1}-\frac{1}{\gamma_{1}}$ and ${\tilde{\gamma }}_{2}={\hat{\gamma }}_{2}-\gamma_{2}$.

### 3.5. Stability Analysis

**Step 1.** On the basis of $\vartheta_{i,s}$, $V_{s}=\sum_{i=1}^{n}\sum_{s=1}^{N}\frac{1}{2}\sigma_{i,s}^{2}$. Deriving from $V_{s}$, one can obtain the following:

(43) $$\begin{array}{l} {\dot{V}}_{s}=\sum_{i=1}^{n}\sum_{s=1}^{N}\sigma_{i,s}\left(F_{i,s}\tau_{i,s}+f_{i,s}\right)\\ \;=\sum_{i=1}^{n}\sum_{s=1}^{N}\left(F_{i,s}\sigma_{i,s}(\Phi_{i,s}(({\hat{\gamma }}_{1,s}+1)\alpha_{i,s})+{℘}_{i,s})+\sigma_{i,s}f_{i,s}\right)\\ \;\leq \sum_{i=1}^{n}\sum_{s=1}^{N}\left(F_{i,s}\sigma_{i,s}\gamma_{1i,s}({\hat{\gamma }}_{1,s}+1)\alpha_{i,s}+F_{i,s}\sigma_{i,s}\gamma_{2i,s}+\sigma_{i,s}f_{i,s}\right) \end{array}$$

**Step 2.** On the basis of ${\hat{\gamma }}_{1}$, $V_{1}=\sum_{i=1}^{n}\sum_{s=1}^{N}\frac{1}{2}\frac{\gamma_{1i,s}}{k_{1,2}}{\tilde{\gamma }}_{1i,s}^{2}$. Deriving from $V_{1}$, one can obtain the following:

(44) $$V_{1}=\sum_{i=1}^{n}\sum_{s=1}^{N}\left(\frac{\gamma_{1i,s}}{k_{1,2}}{\tilde{\gamma }}_{1i,s}\left(-k_{1,1}{\hat{\gamma }}_{1i,s}-F_{i,s}k_{1,2}\sigma_{i,s}\alpha_{i,s}\right)\right)$$

On the basis of Yang’s inequality, one can obtain the following:

(45) $$-{\tilde{\gamma }}_{1i,s}({\tilde{\gamma }}_{1i,s}+\frac{1}{\gamma_{1i,s}})\leq -{\tilde{\gamma }}_{1i,s}^{2}+\frac{1}{2}{\tilde{\gamma }}_{1i,s}^{2}+\frac{1}{2}\frac{1}{\gamma_{1i,s}^{2}}$$

Substituting (45) into (44), one can obtain the following:

(46) $$V_{1}\leq \sum_{i=1}^{n}\sum_{s=1}^{N}\left(-F_{i,s}\sigma_{i,s}\gamma_{1i,s}{\tilde{\gamma }}_{1i,s}\alpha_{i,s}-\frac{1}{2}\frac{k_{1,1}}{k_{1,2}}\gamma_{1i,s}{\tilde{\gamma }}_{1i,s}^{2}+\frac{1}{2}\frac{k_{1,1}}{k_{1,2}}\frac{1}{\gamma_{1i,s}}\right)$$

**Step 3.** On the basis of ${\hat{\gamma }}_{2}$, $V_{2}=\sum_{i=1}^{n}\sum_{s=1}^{N}\frac{1}{2}{\tilde{\gamma }}_{2i,s}^{2}$. Deriving from $V_{2}$, one can obtain the following:

(47) $${\dot{V}}_{2}=\sum_{i=1}^{n}\sum_{s=1}^{N}{\tilde{\gamma }}_{2i,s}\left(-k_{2,1}{\hat{\gamma }}_{2i,s}+\frac{\sigma_{i,s}^{2}F_{i,s}}{\sqrt{\sigma_{i,s}^{2}+k_{2,2}^{2}e^{-2t}}}\right)$$

On the basis of Yang’s inequality, one can obtain the following:

(48) $$-{\tilde{\gamma }}_{2i,s}({\tilde{\gamma }}_{2i,s}+\gamma_{2i,s})\leq -{\tilde{\gamma }}_{2i,s}^{2}+\frac{1}{2}{\tilde{\gamma }}_{2i,s}^{2}+\frac{1}{2}\gamma_{2i,s}^{2}$$

Substituting (48) into (47), one can obtain the following:

(49) $${\dot{V}}_{2}\leq \sum_{i=1}^{n}\sum_{s=1}^{N}\left(\frac{\sigma_{i,s}^{2}F_{i,s}}{\sqrt{\sigma_{i,s}^{2}+k_{2,2}^{2}e^{-2t}}}{\tilde{\gamma }}_{2i,s}-\frac{k_{2,1}}{2}{\tilde{\gamma }}_{2i,s}^{2}+\frac{k_{2,1}}{2}\gamma_{2i,s}^{2}\right)$$

Combining (43), (46) and (49), one can obtain the following:

(50) $$\begin{array}{l} {\dot{V}}_{s}+{\dot{V}}_{1}+{\dot{V}}_{2}\leq \sum_{i=1}^{n}\sum_{s=1}^{N}\left(\begin{array}{l} F_{i,s}\sigma_{i,s}\gamma_{1i,s}({\hat{\gamma }}_{1,s}+1)\alpha_{i,s}-F_{i,s}\sigma_{i,s}\gamma_{1i,s}{\tilde{\gamma }}_{1i,s}\alpha_{i,s}\\ +\frac{\sigma_{i,s}^{2}F_{i,s}}{\sqrt{\sigma_{i,s}^{2}+k_{2,2}^{2}e^{-2t}}}{\tilde{\gamma }}_{2i,s}-\frac{k_{2,1}}{2}{\tilde{\gamma }}_{2i,s}^{2}-\frac{k_{1,1}}{2}\frac{\gamma_{1i,s}}{k_{1,2}}{\tilde{\gamma }}_{1i,s}^{2}\\ +F_{i,s}\sigma_{i,s}\gamma_{2i,s}+\sigma_{i,s}f_{i,s}+\frac{k_{2,1}}{2}\gamma_{2i,s}^{2}+\frac{1}{2}\frac{k_{1,1}}{k_{1,2}}\frac{1}{\gamma_{1i,s}} \end{array}\right)\\ \;\leq \sum_{i=1}^{n}\sum_{s=1}^{N}\left(\begin{array}{l} F_{i,s}\sigma_{i,s}\tau_{hi,s}(1+\gamma_{1i,s})-\gamma_{2i,s}\frac{\sigma_{i,s}^{2}F_{i,s}}{\sqrt{\sigma_{i,s}^{2}+k_{2,2}^{2}e^{-2t}}}+F_{i,s}\sigma_{i,s}\gamma_{2i,s}\\ -\frac{k_{1,1}}{2}\frac{\gamma_{1i,s}}{k_{1,2}}{\tilde{\gamma }}_{1i,s}^{2}-\frac{k_{2,1}}{2}{\tilde{\gamma }}_{2i,s}^{2}+\frac{k_{1,1}}{2k_{1,2}\gamma_{1i,s}}+\frac{k_{2,1}}{2}\gamma_{2i,s}^{2}+\sigma_{i,s}f_{i,s} \end{array}\right) \end{array}$$

On the basis of Lemma 2, one can obtain the following:

(51) $$-\gamma_{2i,s}F_{i,s}\frac{\sigma_{i,s}^{2}}{\sqrt{\sigma_{i,s}^{2}+k_{2,2}^{2}e^{-2t}}}\leq -F_{i,s}\sigma_{i,s}\gamma_{2i,s}+k_{2,2}e^{-t}F_{i,s}\gamma_{2i,s}$$

Substituting (51) into (50), one can obtain the following:

(52) $${\dot{V}}_{s}+{\dot{V}}_{1}+{\dot{V}}_{2}\leq \sum_{i=1}^{n}\sum_{s=1}^{N}\left(\begin{array}{l} F_{i,s}\sigma_{i,s}\tau_{hi,s}(1+\gamma_{1i,s})-\frac{k_{1,1}}{2}\frac{\gamma_{1i,s}}{k_{1,2}}{\tilde{\gamma }}_{1i,s}^{2}-\frac{k_{2,1}}{2}{\tilde{\gamma }}_{2i,s}^{2}\\ +\sigma_{i,s}f_{i,s}+\frac{k_{1,1}}{2k_{1,2}\gamma_{1i,s}}+\frac{k_{2,1}}{2}\gamma_{2i,s}^{2}+k_{2,2}e^{-t}F_{i,s}\gamma_{2i,s} \end{array}\right)$$

From the above, we find that $\tau_{hi,s}$ is an approximate optimal controller derived without considering actuator faults. Therefore, $V_{s0}=\sigma_{i,s}^{2}$, where $\sigma_{i,s}$ does not consider actuator faults.

**Assumption 3**  ([31])**.** *Let $V_{s0}$ be a candidate term of a continuously differentiable Lyapunov function that satisfies the following: *

(53) $${\dot{V}}_{s0}={\left(\nabla V_{s0}(\sigma_{i,s})\right)}^{T}{\dot{\sigma }}_{i,s}={\left(\nabla V_{s0}(\sigma_{i,s})\right)}^{T}(F_{i,s}\tau_{hi,s}+f_{i,s})\leq 0$$

*where $\nabla V_{s0}(\sigma_{i,s})$ is the gradient of $V_{s0}$ across $\sigma_{i,s}$.*

Therefore, there exists a positive definite matrix $\psi_{i,s}\in R^{n\times n}$ satisfying the following:

(54) $${\left(\nabla V_{s0}(\sigma_{i,s})\right)}^{T}((1+\gamma_{1i,s})F_{i,s}\tau_{hi,s}+f_{i,s})\leq -\eta_{\min}(\psi_{i,s})\sigma_{i,s}^{2}$$

Combining (52), (54) and Assumption 3, one can obtain the following:

(55) $${\dot{V}}_{s}+{\dot{V}}_{1}+{\dot{V}}_{2}\leq \sum_{i=1}^{n}\sum_{s=1}^{N}\left(-\eta_{\min}(\psi_{i,s})\sigma_{i,s}^{2}-\frac{k_{1,1}}{2}\frac{\gamma_{1i,s}}{k_{1,2}}{\tilde{\gamma }}_{1i,s}^{2}-\frac{k_{2,1}}{2}{\tilde{\gamma }}_{2i,s}^{2}+\frac{1}{2}\frac{k_{1,1}}{k_{1,2}\gamma_{1i,s}}+\frac{k_{2,1}}{2}\gamma_{2i,s}^{2}+k_{2,2}e^{-t}F_{i,s}\gamma_{2i,s}\right)$$

**Step 4.** On the basis of $\theta_{i,s}$, $V_{3}=\sum_{i=1}^{n}\sum_{s=1}^{N}\frac{1}{2}{\tilde{\theta }}_{i,s}^{2}$. Deriving from $V_{3}$, one can obtain the following:

(56) $${\dot{V}}_{3}=\sum_{i=1}^{n}\sum_{s=1}^{N}{\tilde{\theta }}_{i,s}{\dot{\tilde{\theta }}}_{i,s}=\sum_{i=1}^{n}\sum_{s=1}^{N}\left(\begin{array}{l} -p_{0,s}\frac{\Delta \varphi_{i,s}^{T}\Delta \varphi_{i,s}}{{\left(1+\Delta \varphi_{i,s}^{T}\Delta \varphi_{i,s}\right)}^{2}}{\tilde{\theta }}_{i,s}^{2}-p_{0,s}\sum_{l=1}^{L}\frac{\Delta \varphi_{i,s}^{lT}\Delta \varphi_{i,s}^{l}}{{\left(1+\Delta \varphi_{i,s}^{lT}\Delta \varphi_{i,s}^{l}\right)}^{2}}{\tilde{\theta }}_{i,s}^{2}+\\ p_{0,s}\frac{\Delta \varphi_{i,s}}{{\left(1+\Delta \varphi_{i,s}^{T}\Delta \varphi_{i,s}\right)}^{2}}\Delta \xi_{i,s}{\tilde{\theta }}_{i,s}+p_{0,s}\sum_{l=1}^{L}\frac{\Delta \varphi_{i,s}^{l}}{{\left(1+\Delta \varphi_{i,s}^{lT}\Delta \varphi_{i,s}^{l}\right)}^{2}}\Delta \xi_{i,s}^{l}{\tilde{\theta }}_{i,s} \end{array}\right)$$

On the basis of Yang’s inequality, one can obtain the following:

(57) $$\frac{\Delta \varphi_{i,s}}{{\left(1+\Delta \varphi_{i,s}^{T}\Delta \varphi_{i,s}\right)}^{2}}\Delta \xi_{i,s}{\tilde{\theta }}_{i,s}\leq \frac{1}{2}\frac{\Delta \varphi_{i,s}^{T}\Delta \varphi_{i,s}}{{\left(1+\Delta \varphi_{i,s}^{T}\Delta \varphi_{i,s}\right)}^{4}}{\tilde{\theta }}_{i,s}^{2}+\frac{1}{2}\Delta \xi_{i,s}^{2}$$

(58) $$\sum_{l=1}^{L}\frac{\Delta \varphi_{i,s}^{l}}{{\left(1+\Delta \varphi_{i,s}^{lT}\Delta \varphi_{i,s}^{l}\right)}^{2}}\Delta \xi_{i,s}^{l}{\tilde{\theta }}_{i,s}\leq \frac{1}{2}\sum_{l=1}^{L}\frac{\Delta \varphi_{i,s}^{lT}\Delta \varphi_{i,s}^{l}}{{\left(1+\Delta \varphi_{i,s}^{lT}\Delta \varphi_{i,s}^{l}\right)}^{4}}{\tilde{\theta }}_{i,s}^{2}+\frac{1}{2}\sum_{l=1}^{L}\Delta \xi_{i,s}^{l2}$$

Combining (56)–(58), one can obtain the following:

(59) $$\varphi_{\theta }=\frac{\Delta \varphi_{i,s}^{T}\Delta \varphi_{i,s}}{{\left(1+\Delta \varphi_{i,s}^{T}\Delta \varphi_{i,s}\right)}^{2}}-\frac{1}{2}\frac{\Delta \varphi_{i,s}^{T}\Delta \varphi_{i,s}}{{\left(1+\Delta \varphi_{i,s}^{T}\Delta \varphi_{i,s}\right)}^{4}}=\frac{\Delta \varphi_{i,s}^{T}\Delta \varphi_{i,s}2({\left(1+\Delta \varphi_{i,s}^{T}\Delta \varphi_{i,s}\right)}^{2}-1)}{2{\left(1+\Delta \varphi_{i,s}^{T}\Delta \varphi_{i,s}\right)}^{4}}>0$$

(60) $$\varphi_{\theta }^{L}=\sum_{l=1}^{L}\frac{\Delta \varphi_{i,s}^{lT}\Delta \varphi_{i,s}^{l}}{{\left(1+\Delta \varphi_{i,s}^{lT}\Delta \varphi_{i,s}^{l}\right)}^{2}}-\frac{1}{2}\sum_{l=1}^{L}\frac{\Delta \varphi_{i,s}^{lT}\Delta \varphi_{i,s}^{l}}{{\left(1+\Delta \varphi_{i,s}^{lT}\Delta \varphi_{i,s}^{l}\right)}^{4}}=\sum_{l=1}^{L}\frac{\Delta \varphi_{i,s}^{lT}\Delta \varphi_{i,s}^{l}2({\left(1+\Delta \varphi_{i,s}^{lT}\Delta \varphi_{i,s}^{l}\right)}^{2}-1)}{2{\left(1+\Delta \varphi_{i,s}^{lT}\Delta \varphi_{i,s}^{l}\right)}^{4}}>0$$

Combining (56)–(60), one can obtain the following:

(61) $${\dot{V}}_{3}\leq \sum_{i=1}^{n}\sum_{s=1}^{N}\left(-p_{0,s}(\varphi_{\theta i,s}+\varphi_{\theta i,s}^{L}){\tilde{\theta }}_{i,s}^{2}+\frac{1}{2}p_{0,s}(\Delta \xi_{i,s}^{2}+\sum_{l=1}^{L}\Delta \xi_{i,s}^{l2})\right)$$

**Step 5.** On the basis of $C_{i,s}^{*}(\sigma_{i,s})$, $V_{4}=\sum_{i=1}^{n}\sum_{s=1}^{N}C_{i,s}^{*}(\sigma_{i,s})$. Deriving from $V_{5}$, one can obtain the following:

(62) $${\dot{V}}_{4}=\sum_{i=1}^{n}\sum_{s=1}^{N}{\dot{C}}_{i,s}^{*}(\sigma_{i,s})=\sum_{i=1}^{n}\sum_{s=1}^{N}\left(wC_{i,s}^{*}-N_{i,s}(\sigma_{i,s},\tau_{hi,s}^{*})\right)\leq \sum_{i=1}^{n}\sum_{s=1}^{N}\left(-\sigma_{i,s}^{2}+wC_{i,s}^{*}\right)$$

**Step 6.** On this basis $V=V_{s}+V_{1}+V_{2}+V_{3}+V_{4}$. Deriving from V, one can obtain the following:

(63) $$\begin{array}{l} \dot{V}\leq \sum_{i=1}^{n}\sum_{s=1}^{N}\left(\begin{array}{l} -\eta_{\min}(\psi_{i,s})\sigma_{i,s}^{2}-\frac{k_{1,1}}{2}\frac{\gamma_{1i,s}}{k_{1,2}}{\tilde{\gamma }}_{1i,s}^{2}-\frac{k_{2,1}}{2}{\tilde{\gamma }}_{2i,s}^{2}+\frac{1}{2}\frac{k_{1,1}}{k_{1,2}\gamma_{1i,s}}+\frac{k_{2,1}}{2}\gamma_{2i,s}^{2}+\gamma_{2i,s}k_{2,2}e^{-t}F_{i,s}\\ -p_{0,s}(\varphi_{\theta }+\varphi_{\theta }^{L}){\tilde{\theta }}_{i,s}^{2}+\frac{1}{2}p_{0,s}(\Delta \xi_{i,s}^{2}+\sum_{l=1}^{L}\Delta \xi_{i,s}^{l2})-\sigma_{i,s}^{2}+wC_{i,s}^{*} \end{array}\right)\\ \;\leq \sum_{i=1}^{n}\sum_{s=1}^{N}\left(-\mu_{1}\left\|x_{i,s}\right\|+\mu_{2}\right) \end{array}$$

where $x_{i,s}={\left[\sigma_{i,s},{\tilde{\gamma }}_{1i,s},{\tilde{\gamma }}_{2i,s},{\tilde{\theta }}_{i,s}\right]}^{T}$, $\mu_{1}=\min(\eta_{\min}(\psi_{i,s})+1,\frac{\gamma_{1i,s}k_{1,1}}{2k_{1,2}},\frac{k_{2,1}}{2},p_{0,s}(\varphi_{\theta }+\varphi_{\theta }^{L}))$ and $\mu_{2}=\frac{1}{2}\frac{k_{1,1}}{k_{1,2}\gamma_{1i,s}}+\frac{k_{2,1}}{2}\gamma_{2i,s}^{2}+\gamma_{2i,s}k_{2,2}e^{-t}F_{i,s}+\frac{1}{2}p_{0,s}(\Delta \xi_{i,s}^{2}+\sum_{l=1}^{L}\Delta \xi_{i,s}^{l2})+wC_{i,s}^{*}>0$.

Finally, one can obtain the following:

(64) $$\dot{V}\leq 0,\forall \left\|x_{i,s}\right\|\geq \frac{\mu_{2}}{\mu_{1}}$$

On the basis of the standard Lyapunov extension lemma of [44], the trajectories of the multiple robotic manipulators are verified to be uniformly ultimately bounded (UUB).

**Proof.**  On the basis of assumption 2 and the $e^{-w(r-t)}$ term, the cost function approximation cannot be infinite in a real system even though the tracking error does not eventually converge to 0. On the basis of Section 2.4, the critic-only NN approximation error and its gradient are both bounded. It is simple to obtain $\Delta \xi_{i,s}=\xi_{i,s}(t_{1})-e^{-wT_{w}}\xi_{i,s}\leq 2{\bar{\xi }}_{i,s}$ and $\Delta \xi_{i,s}^{l}=\xi_{i,s}^{l}(t_{1})-e^{-wT_{w}}\xi_{i,s}^{l}\leq 2{\bar{\xi }}_{i,s}^{l}$. On the basis of Assumption 1, the actuator fault influences are bounded. For equation $F_{i,s}=b_{2}u_{i,s}H_{ti,s}$, Lemma 2 shows that $H_{ti,s}$ is bounded, it is obvious from $b_{2i,s}=\frac{a_{2}}{{\left(2-{\left(\frac{2}{\pi }\arctan(E_{i,s})\right)}^{2}\right)}^{2c_{2}}}$ that $\frac{a_{2}}{2^{2c_{2}}}\leq b_{2i,s}\leq a_{2}$, and it is obvious from $r_{i,s}=\sec^{2}(\frac{\arctan(E_{i,s})}{\beta_{i,s}})\frac{1}{\beta_{i,s}(1+E_{i,s}^{2})}$ that $0<r_{i,s}\leq \sec^{2}(\frac{\pi }{2\beta_{i,s}})\frac{1}{\beta_{i,s}}$. It can be concluded that $F_{i,s}$ is bounded. Assumption 1 and Assumption 2 can be fulfilled in practice. In summary, we can consider $\mu_{2}$ to be bounded. Therefore, the UUB of the multiple robotic manipulator system can be ensured under the control scheme of this paper. The proof ends. □

**Remark 8.**  *Since the critic-only NN is related to the estimation error, it can guarantee only UUB stability for cooperative control with multiple robotic manipulators. However, one can obtain the desired control performance by adjusting the design parameters of the controller and choosing the appropriate neural network structure.*

## 4. Simulations

### 4.1. Simulation Conditions

**Figure 2:**
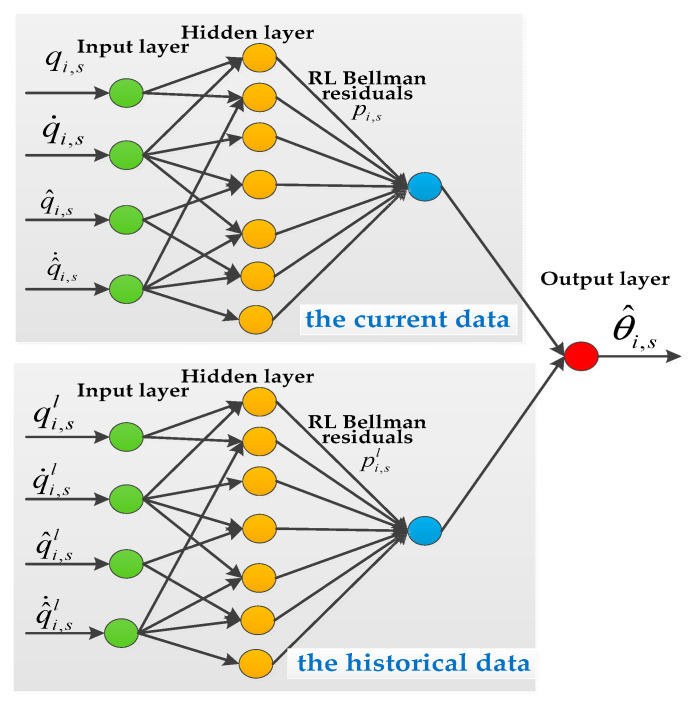
The critic-only NN structure.

**Figure 3:**
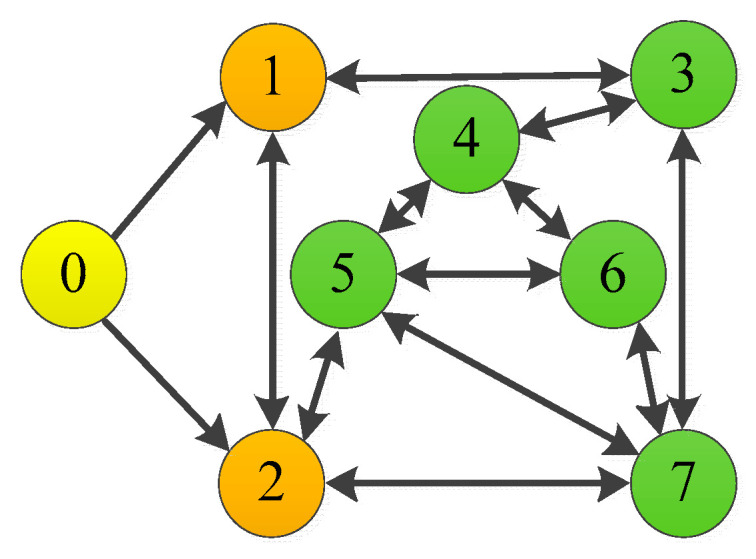
Interaction of multiple robotic manipulators with two degrees of freedom.

**Figure 4:**
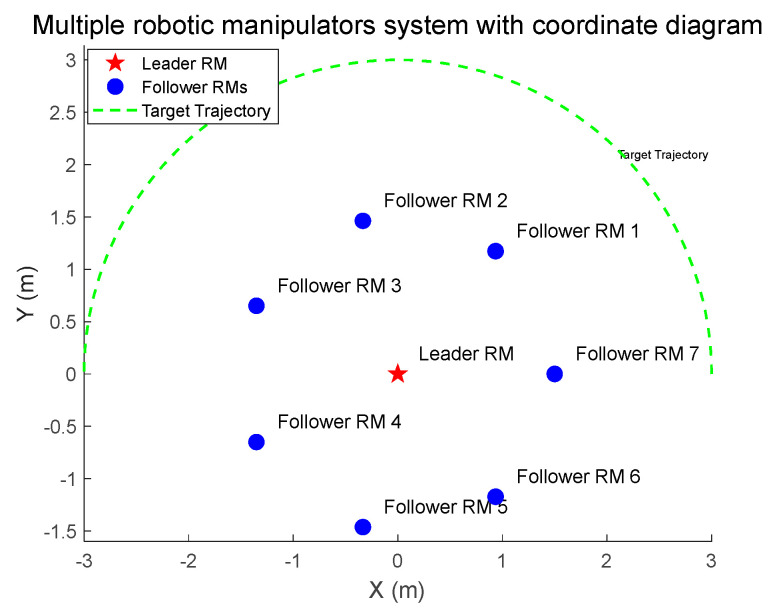
Multiple robotic manipulator systems with a coordinate diagram.

In this section, multiple two-degree-of-freedom manipulator systems consisting of a leader and seven followers are demonstrated. The dynamics model is given by the following entries:

$$\begin{array}{l} M_{i}=\left[\begin{array}{l} P_{i,1}+2P_{i,2}\cos(q_{i,2})\;P_{i,3}+P_{i,2}\cos(q_{i,2})\\ P_{i,3}+P_{i,2}\cos(q_{i,2})\;P_{i,3} \end{array}\right]\\ C_{i}=\left[\begin{array}{l} -P_{i,2}\sin(q_{i,2}){\dot{q}}_{i,1}\;-P_{i,2}\sin(q_{i,2})({\dot{q}}_{i,1}+{\dot{q}}_{i,2})\\ P_{i,2}\sin(q_{i,2}){\dot{q}}_{i,2}\;0 \end{array}\right] \end{array}$$

where $p_{i,1}=J_{i,1}+m_{i,1}r_{i,a1}^{2}+m_{i,2}r_{i,1}^{2}+J_{i,2}+m_{i,2}r_{i,a2}^{2}$; $p_{i,2}=m_{i,2}r_{i,1}r_{i,a2}$; $p_{i,3}=J_{i,2}+m_{i,2}r_{i,a2}^{2}$; $m_{i,1}$ and $m_{i,2}$ denote the mass of the robotic manipulator; $J_{i,1}$ and $J_{i,2}$ denote the moment of inertia of the robotic manipulator; $r_{i,1}$ and $r_{i,2}$ denote the length of the robotic manipulator; and $r_{i,a1}$ and $r_{i,a2}$ denote the center of mass of the robotic manipulator. The critic-only NN structure is shown in Figure 2. The interactive topology of leaders and followers is shown in Figure 3. The multiple robotic manipulator system with a coordinate diagram is shown in Figure 4. The multiple manipulator model parameters are shown in Table 2. The system’s parameters and initial conditions are shown in Table 3.

**Remark 9.** 
*To explain the effects of various parameter choices on the performance of the control system, the following basic suggestions for parameter adjustment are given:*

(1) For the dynamic distributed observer (DDO), the selection of parameter $g_{c}(c=1,2,3,4)$ needs to balance the observation accuracy and computational efficiency: increasing $g_{c}(c=1,2,3,4)$ can reduce the observation error of robotic manipulators and improve the control accuracy, but it increases the computational burden and affects the real-time performance. The value of $g_{c}(c=1,2,3,4)$ should be optimized in practical applications to ensure sufficient accuracy while avoiding excessive consumption of computational resources to achieve the best trade-off between error and computational cost.

(2) For the QPPC method, the initial value ($\beta_{oi,s}$) plays a role in determining the initial error bounds and has an impact on the transient performance; the final value ($\beta_{\infty i,s}$) affects the ultimate convergence performance; the convergence time ($T_{a}$) necessitates a careful balance between velocity, which favors small values, and stability, which requires large values.

(3) For the AGITSMC method, as fundamental proportionality coefficients, $a_{1}$ and $a_{2}$ directly establish the benchmark for the magnitude values of $b_{1i,s}$ and $b_{2i,s}$, meaning that larger values of $a_{1}$ and $a_{2}$ will correspondingly increase the overall magnitude values of $b_{1i,s}$ and $b_{2i,s}$, which in turn affects the calculation of the sliding surface and influences the dynamic performance of the control system; as small positive parameters, $c_{1}$ and $c_{2}$ dictate the change rates of $b_{1i,s}$ and $b_{2i,s}$ with respect to variations in $E_{i,s}$ and $e_{i,s}$, where larger $c_{1}$ and $c_{2}$ values heighten the sensitivity of functions $b_{1i,s}$ and $b_{2i,s}$ to such changes, enabling quicker adjustments of $b_{1i,s}$ and $b_{2i,s}$ and thus facilitating a more prompt system response to error changes; $b_{3}$ is a positive number that can balance the impact of current errors and historical errors on the sliding surface.

(4) For the CNNODP method, feedback gains ($\lambda_{i,s}$) optimize the system response by balancing the convergence velocity and stability, increasing the degree of convergence, but excessive gain causes oscillation; the discount factor (w > 0) serves to strike a balance between the significance of the present performance and future outcomes while simultaneously enhancing computational stability by preventing the unrestricted accumulation of future costs or rewards in the control system; the learning rate ($p_{0,s}$) is crucial in the gradient descent algorithm, as it controls the step size, influences the convergence speed and stability, and balances speed with accuracy to ensure effective convergence to a good solution; ($T_{w}$) usually represents a time interval that balances the system’s consideration of current and future performance by defining the integral interval, influencing the stability of the optimization results, and thereby achieving more effective control optimization; the experience sample (L) provides diverse data to facilitate comprehensive system understanding, smooths the update process, boosts computational efficiency, and prevents overfitting to recent experiences.

(5) For the fault compensation control method, as a positive parameter, ($k_{1,1}$, $k_{1,2}$, $k_{2,1}$, and $k_{2,2}$) control the rate of compensation of the fault compensation rate, which is greater for a faster response to changes in actuator faults and smaller for slower and more conservative updating; thus, it needs to be carefully adjusted to ensure stable and accurate fault compensation. ($k_{1,1}$, $k_{1,2}$, $k_{2,1}$, and $k_{2,2}$) are positive numbers that control the compensation speed of the fault compensation rate. The larger the values of ($k_{1,1}$, $k_{1,2}$, $k_{2,1}$, and $k_{2,2}$ are, the faster the fault compensation rate responds to changes in actuator faults, and a smaller value results in a slower and more conservative update. These parameters need to be carefully adjusted to ensure the stability and accuracy of fault compensation.

### 4.2. Simulation Analysis

**Figure 5:**
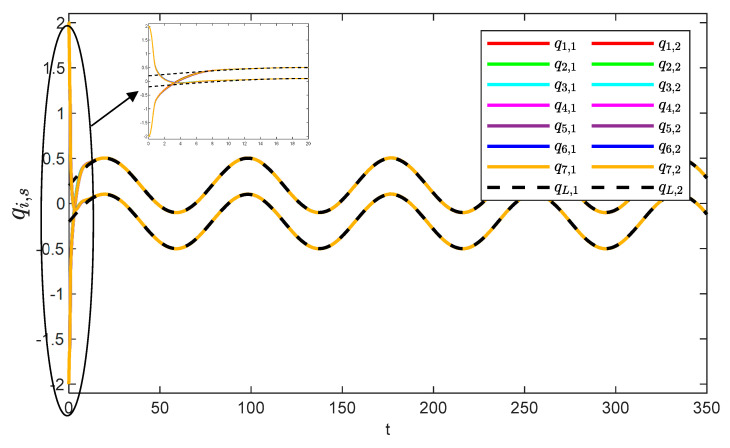
Angular consistency effect.

**Figure 6:**
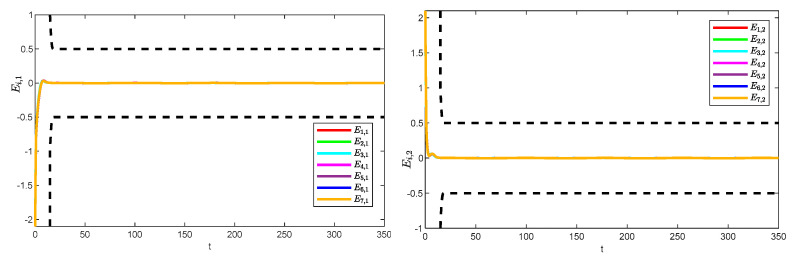
Trajectory errors of the system.

**Figure 7:**
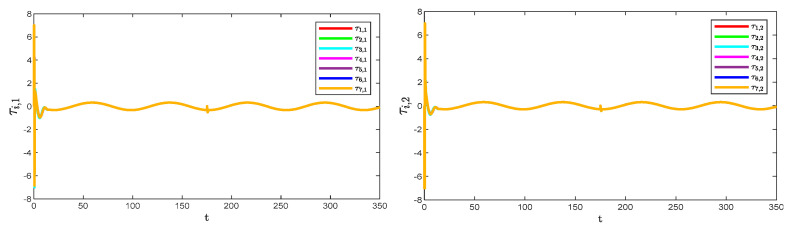
Control inputs to the system.

Figure 5 and Figure 6 show the position tracking and tracking error profiles for one leader and seven followers via the QPPC strategy. Figure 7 illustrates the control inputs of the robotic manipulator. Specifically, the system is unconstrained from the initial period to a certain time period, i.e., when $\beta_{i,s}\geq 1$, and the system error is constrained to a predefined time domain when $\beta_{i,s}<1$. Therefore, Figure 5 and Figure 6 show that the designed controller realizes flexible performance constraints and has good tracking performance throughout. Figure 6 shows the control inputs of the multiple robotic manipulators. The control inputs eventually converge and remain bounded and very stable without significant jitter.

**Figure 8:**
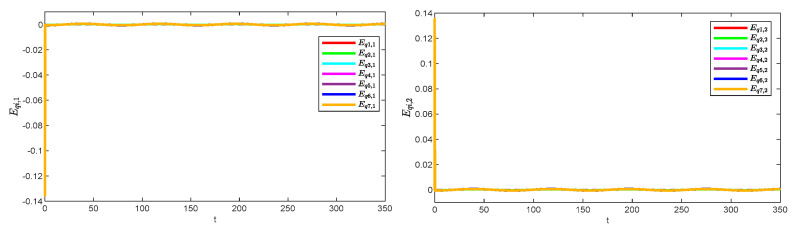
Observation errors of the system.

**Figure 9:**
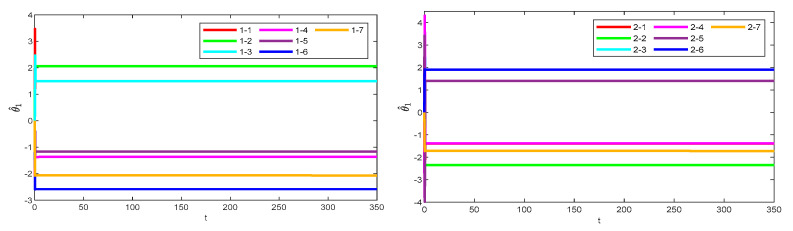
Learning process of the critic-only NN weights for robotic manipulator 1.

Figure 8 illustrates the observation errors for the multiple robotic manipulators. The observation errors eventually converge and are always bounded. Figure 9 shows the convergence process of the critic-only NN weight estimation for robotic manipulator 1, which ultimately remains stable. The critic-only NN weight estimation eventually converges and eventually remains stable. Therefore, optimal control is achieved by using the critic-only strategy, which is approximately one-half the computation of the actor–critic strategy [22,23,24] and one-third of the identifier–actor–critic strategy [25,26].

### 4.3. Comparative Simulation

#### 4.3.1. Comparative Simulation for the AGITSMC Control Strategy

To highlight the advantages of the AGITSMC control strategy, comparisons with those of [11,12,13] are given.

**Case 1** ([13]).
  (65) $$\vartheta_{i,s}={\dot{E}}_{i,s}+k_{1}\frac{{\left|\rho_{i}\right|}^{k_{1}}}{{\left|\rho_{i}\right|}^{k_{2}}-{\left|E_{i,s}\right|}^{k_{2}}}sig^{k_{3}}(E_{i,s})$$
  *where* $k_{1}$ *,* $k_{2}>1$ *and* $k_{3}>1$ *are positive parameters,* *and* $\rho_{i}$ *is the prescribed performance function.*

**Case 2** ([12]).
  (66) $$\vartheta_{i,s}={\dot{E}}_{i,s}+k_{4}\int_{0}^{t}sig^{k_{5}}(E_{i,s})dt$$
  *where* $k_{4}$ *and* $k_{5}$ *are positive parameters.*

**Case 3** ([11]).
  (67) $$\vartheta_{i,s}=E_{i,s}+k_{6}sig^{k_{7}}(E_{i,s})+k_{8}sig^{k_{9}}({\dot{E}}_{i,s})$$
  *where* $k_{6}$ *,* $k_{7}$ *,* $k_{8}$ *and* $k_{9}$ *are positive parameters.*

**Figure 10:**
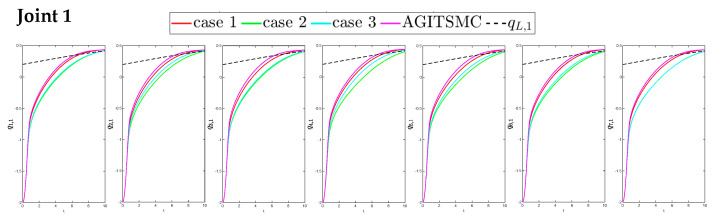
Comparison of trajectory convergence velocities from 0 to 10 s for the four strategies.

**Figure 11:**
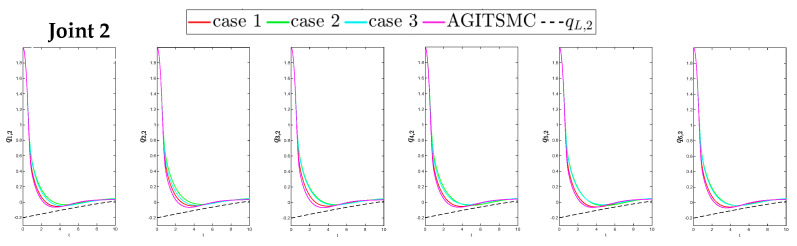
Comparison of trajectory convergence velocities from 0 to 10 s for the four strategies.

**Figure 12:**
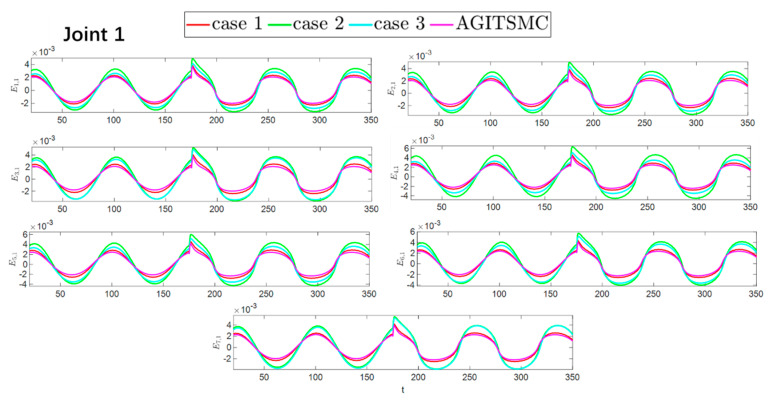
Comparison of the error convergence domain at 20–350 s for the four strategies.

**Figure 13:**
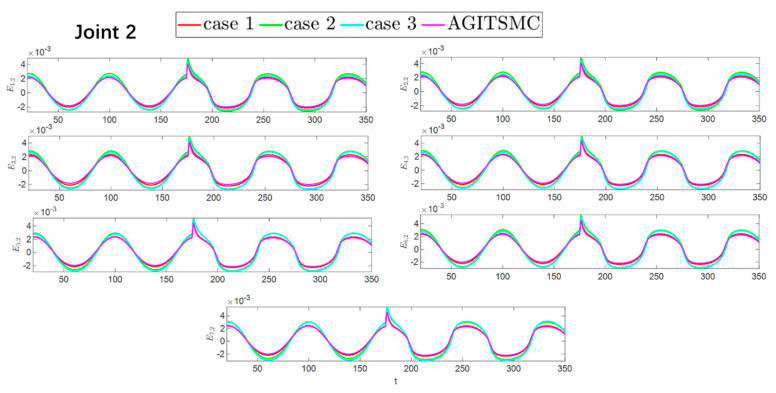
Comparison of the error convergence domain at 20–350 s for the four strategies.

**Figure 14:**
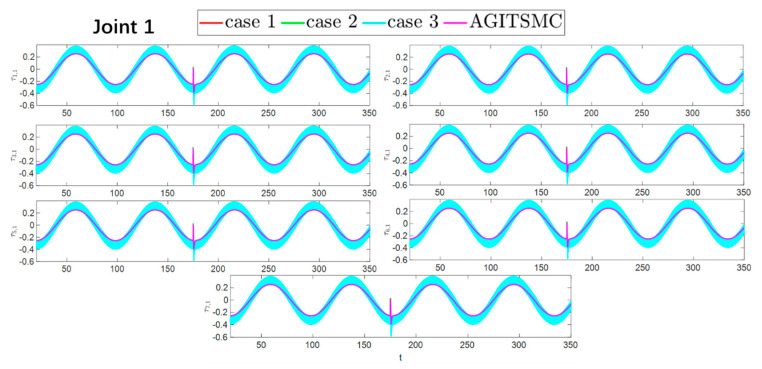
Comparison of control inputs in the range of 20–350 s for the four strategies.

**Figure 15:**
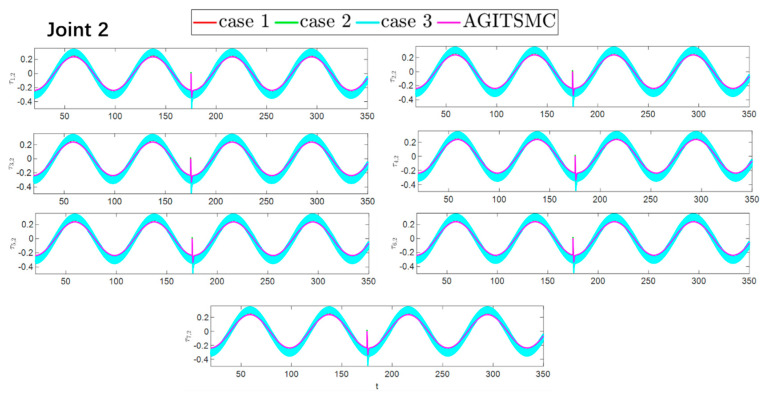
Comparison of control inputs in the range of 20–350 s for the four strategies.

To better show the differences in the control performance of the four strategies, local comparisons of the trajectory convergence velocity, error convergence domain, and control inputs for the four control strategies are presented. The parameters for the comparative experiments are shown in Table 4. Figure 10 and Figure 11 show a comparison of the trajectory convergence velocities from 0 to 10 s for the four control strategies. From the two figures. The proposed AGITSMC strategy has the fastest convergence velocity. Figure 12 and Figure 13 show the comparisons of the error convergence domain in the 20–350 s range for the four control strategies. The two figures show that the tracking errors of the four strategies are within a predefined domain, especially after the actuator faults are within a predefined domain. Therefore, the four strategies have good steady-state performance. Specifically, among the four control strategies, the AGITSMC strategy has the smallest error convergence domain and the best tracking effect. Figure 14 and Figure 15 show comparisons of the control inputs from 20 to 350 s for the four strategies. The control inputs of the proposed AGITSMC strategy are much smoother and recover to a smooth state even after actuator faults. In contrast, the control inputs of the other three strategies have a certain level of jitter, especially in case 3, which obviously cannot address the problem of input jitter well. In addition, the control energy of the AGITSMC strategy is the smallest.

To fully evaluate the effectiveness of the four strategies, three indicators are used to quantify their performance. They are the integral of the absolute value of the error (IAE), the integral of the time multiplied by the absolute value of the error (ITAE), and the integral of the square value (ISV) of the control input, which are described as follows:

(68) $$\left\{\begin{array}{l} IAE=\int_{0}^{350}\left|E_{i,s}\right|dt\\ ITAE=\int_{0}^{350}t\left|E_{i,s}\right|dt\\ ISV=\int_{0}^{350}\tau_{i,s}^{2}dt \end{array}\right.$$

To facilitate the comparative performance indicators of the seven robotic manipulators in the four strategies, we directly take the average of the corresponding indicators of the seven robotic manipulators. The relevant data are summarized in Table 5. Table 5 shows that both the IAE and ITAE indicators of the proposed AGITSMC are minimized, which means that the AGITSMC has a smaller tracking error and higher dynamic tracking accuracy than the comparative strategies do; the ISV indicator of the AGITSMC is minimized, which means that the control cost of the proposed controller is minimized. Thus, compared with existing SMC strategies, the proposed AGITSMC not only offers better tracking performance but also saves more cost.

#### 4.3.2. Comparative Simulation for the CNNODP Control Strategy

To highlight the advantages of the CNNODP control strategy, comparisons with the actor–critic method [22,23,24] are given.

**Figure 16:**
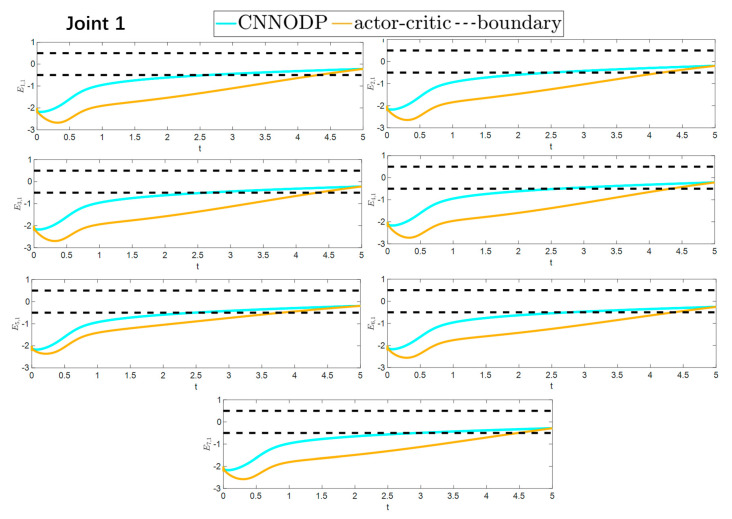
Comparison of the convergence velocity for the two strategies.

**Figure 17:**
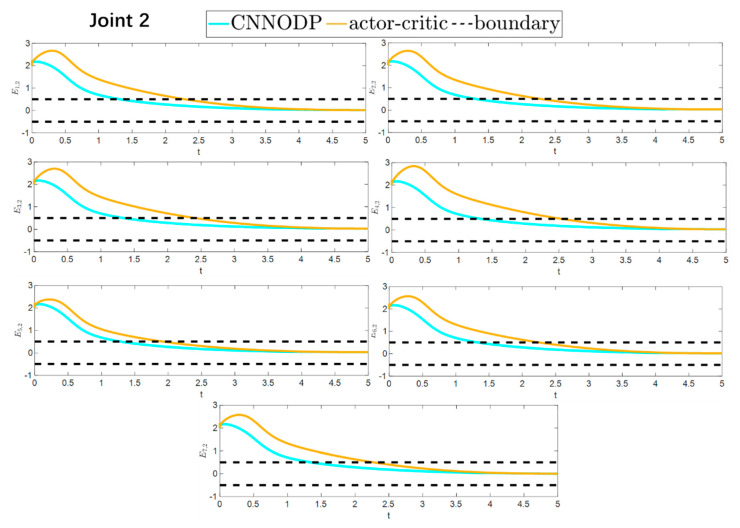
Comparison of the convergence velocity for the two strategies.

**Figure 18:**
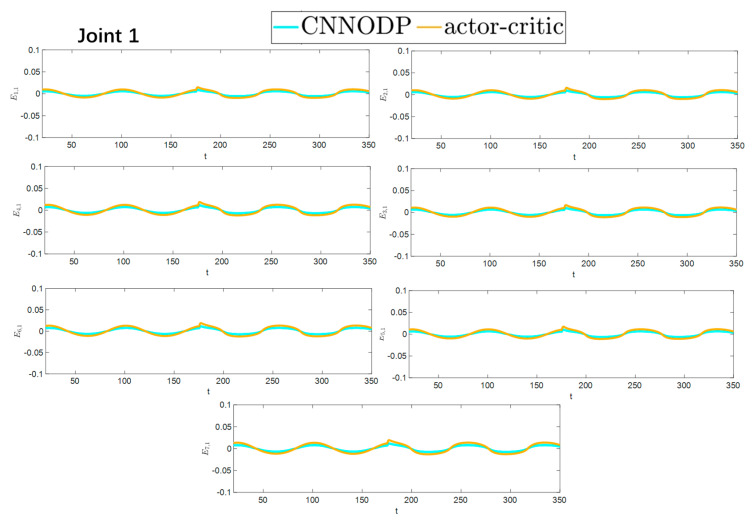
Comparison of the convergence domain for the two strategies.

**Figure 19:**
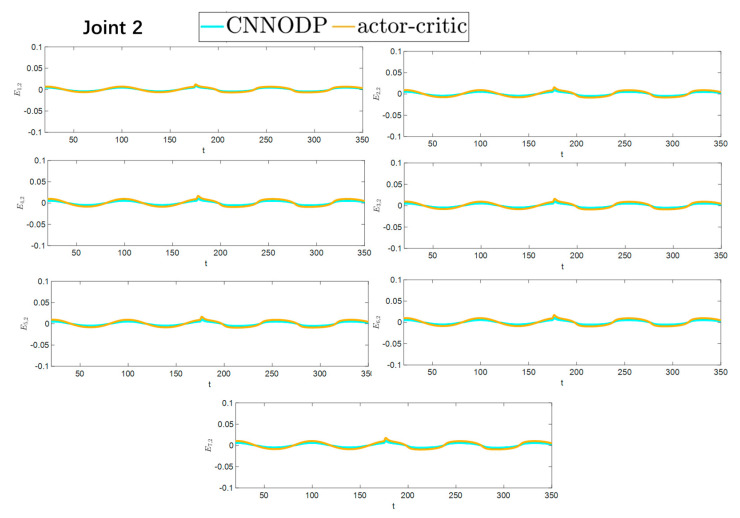
Comparison of the convergence domain for the two strategies.

The convergence speeds of the two strategies are shown in Figure 16 and Figure 17, indicating that the CNNODP strategy converges significantly faster. This is because CNNODP only needs to optimize a single value network without the coupled update issues between the actor and critic networks. In simple state-action mapping scenarios, it can quickly learn effective control rules, leading to a faster error reduction rate than the more complex actor–critic architecture can achieve. Figure 18 and Figure 19 clearly show a comparison of the error convergence domains of the two strategies. The results clearly reveal that the convergence error of the CNNODP strategy is significantly smaller. The core reason for this advantage lies in the fact that the value function design of the CNNODP strategy enables it to directly learn the “optimal action-state matching” rules without going through complex network interaction processes. Therefore, unlike other strategies, it does not generate additional deviations because of network coupling issues. This characteristic allows the CNNODP strategy to maintain high tracking accuracy even after the system stabilizes.

## 5. Conclusions

In this paper, adaptive optimal sliding mode fault-tolerant control is proposed for multiple robotic manipulators on the basis of quantitative prescribed performance control and critic-only dynamic programming. The QPPC strategy releases the initial state of the control system to realize global predetermined performance control. The AGITSMC strategy not only improves the convergence velocity and control accuracy but also reduces the jitter of the system. The CNNODP strategy not only reduces the computational effort but also achieves optimal control. The adaptive fault compensation control strategy addresses actuator faults. The simulation results of the control strategies constructed in this paper under external disturbances and actuator faults and the comparison of the simulation results with those of the existing methods have proven the effectiveness of the proposed strategy. The simulation results show that the proposed control strategies have very good control performance. Specifically, the QPPC strategy divides the error constraint into two parts, i.e., when $\beta_{i,s}\geq 1$, the system is unconstrained and when $\beta_{i,s}<1$, the system is constrained to a predefined domain, and the control system retains a very good tracking effect throughout the whole process. Compared with other existing strategies, the AGITMC strategy ensures faster convergence of the system, less control cost, and the best interference ability. The learning law of the CNNODP strategy remains stable throughout the process, with a good learning effect. In conclusion, the proposed control strategy can achieve optimal robust control of multiple robotic manipulators under external disturbances and actuator faults. From the perspective of research development, it is necessary to further expand the section on future work, and conducting tests in different fields has numerous positive implications. For example, in various scenarios of industrial automation production, testing the applicability of this method to different types of multiple robotic manipulator systems can further validate its robustness and versatility. In the aerospace field, when confronted with complex and high-precision operational tasks as well as harsh environmental conditions, testing this method can explore its performance under extreme circumstances. Therefore, if tests can be carried out in different fields in the future, they will contribute to a more comprehensive evaluation of the value and application prospects of this method.

## Figures and Tables

**Table 1:** Abbreviated table.

Parameters	Significance	Parameters	Significance
$q_{i,s}$	position	${\mathbb{C}}_{i,s}$	performance index
${\dot{q}}_{i,s}$	velocity	$N_{i,s}$	cost function
${\ddot{q}}_{i,s}$	acceleration vectors	$H_{i,s}$	Hamilton–Jacobi–Bellman
$M_{i}$	inertia matrix	$C_{i,s}^{0}$	unknown continuous function
$C_{i}$	centripetal and Coriolis force term	$\tau_{i,s}^{*}$/${\hat{\tau }}_{i,s}$	ideal/estimation of control input
$G_{i}$	gravity vector	$C_{i,s}^{0}$	unknown continuous function
$\tau_{i,s}$	input torque vectors	$E_{i,s}$	angle error
$\Gamma_{i}$	external disturbance	$\xi_{i,s}$	approximation error
$\tau_{hi,s}$	input of fault model	$\varphi_{i,s}$	basis function vector
$\Phi_{i,s}$	bias fault of actuator	$p_{i,s}$	RL Bellman residuals
${℘}_{i,s}$	additive fault of actuator	${\hat{\gamma }}_{1}$/${\hat{\gamma }}_{2}$	adaptive parameters
$q_{L,s}$	leader’s trajectory	V	Lyapunov function
$v_{0}$	leader’s velocity	$a_{ij}$	communication among agents
$h_{0}$	leader’s acceleration	$\beta_{i,s}$	auxiliary function
${\hat{q}}_{i,s}$	follower’s observer trajectory	$Z_{i,s}$	conversion error
$v_{i,s}$	follower’s observer velocity	$\sigma_{i,s}$	sliding mode variable
$h_{i,s}$	follower’s observer acceleration	ℜ	set of real numbers
θ/$\hat{\theta }$/$\tilde{\theta }$	ideal/estimation/error of adaptive parameters	$b_{i,s}$	communication among leader and agents
$E_{ti,s}$	quantization error	$\left\|*\right\|$	Euclidean norm
${ℜ}^{n}$	n-dimensional Euclidean space	${ℜ}^{n\times n}$	n×n-dimensional Euclidean space
I	identity matrix		

**Table 2:** Model parameters of multiple robotic manipulators.

**RMS**	$\frac{J_{i,1}}{kg\cdot m^{2}}$	$\frac{J_{i,2}}{kg\cdot m^{2}}$	$\frac{m_{i,1}}{kg}$	$\frac{m_{i,2}}{kg}$	$\frac{r_{i,1}}{m}$	$\frac{r_{i,2}}{m}$	$\frac{r_{i,a1}}{m}$	$\frac{r_{i,a2}}{m}$
**R-1**	0.6	0.8	1.5	2.0	1.7	1.5	2.2	2.8
**R-2**	0.5	0.6	1.1	1.2	1.8	1.5	2.2	2.8
**R-3**	0.6	0.7	2.1	2.0	2.9	2.3	1.0	0.8
**R-4**	0.8	0.9	2.0	1.9	2.6	2.6	1.3	0.9
**R-5**	0.7	0.8	3.9	3.0	3.7	3.4	3.1	3.8
**R-6**	0.5	0.6	3.2	3.7	3.8	3.8	3.4	3.9
**R-7**	0.9	0.9	2.8	2.1	2.6	2.8	2.3	1.8

**Table 3:** Initial parameters and initial conditions of the system.

**System state**	$q_{L}=[0.3\sin(0.08t)+0.2,0.3\sin(0.08t){-0.2]}^{T}$, $G_{i}(q_{i})={\left[9.8,9.8\right]}^{T}$, ${\hat{q}}_{i}(0)=[0.2,{-0.2]}^{T}$, $q_{i}{\left(0)=[-2,2\right]}^{T}$, ${\dot{q}}_{i}(0)={\left[0,0\right]}^{T}$, ${\ddot{q}}_{i}(0)={\left[0,0\right]}^{T}$, $v_{i}(0)={\left[0,0\right]}^{T}$, $h_{i}(0)={\left[0,0\right]}^{T}$, $\Gamma_{i}=[0.25\sin3t,0.25{\sin3t]}^{T}$
**Observer**	$g_{1}=5$, $g_{2}=4$, $g_{3}=7$, $g_{4}=8$
**QPPC**	$\beta_{oi,s}=10$, $\beta_{\infty i,s}=0.5$, $T_{a}=20$
**AGITSM**	$a_{1}=10$, $a_{2}=7$, $b_{3}=0.2$, $c_{1}=2$, $c_{2}=2$
**CNNODP**	$\varphi_{i,s}={\left[0.1q_{i,s}{\dot{q}}_{i,s}q_{i,s}{\dot{\hat{q}}}_{i,s}\;0.1{\dot{q}}_{i,s}{\dot{q}}_{i,s}\;0.1{\dot{q}}_{i,s}{\hat{q}}_{i,s}\;0.1{\dot{q}}_{i,s}{\dot{\hat{q}}}_{i,s}{\hat{q}}_{i,s}{\dot{\hat{q}}}_{i,s}{\dot{\hat{q}}}_{i,s}{\dot{\hat{q}}}_{i,s}\right]}^{T}$, $\lambda_{i,s}=10$, $p_{0,s}=1$, w=0.1, $T_{w}=0.05$, L=10
**Fault control**	${℘}_{i,s}=0.05+0.05\sin(0.8t)$, $k_{1,1}=0.1$, $k_{1,2}=50$, $k_{2,1}=0.1$, $k_{2,2}=20$, $\Phi_{i,s}=0.01$, ${\hat{\gamma }}_{1i,s}(0)={\left[0,0\right]}^{T}$, ${\hat{\gamma }}_{2i,s}(0)={\left[0,0\right]}^{T}$

**Table 4:** The parameters of the comparative experiment.

Case	Case 1	Case 2	Case 3
**Parameters**	$k_{1}=7$, $k_{2}=k_{3}=2$	$k_{4}=0.2$, $k_{5}=2$	$k_{6}=9$, $k_{8}=7$, $k_{7}=k_{9}=2$

**Table 5:** Comparison of three control performance indicators for the four strategies.

Strategies	IAE		ITAE		ISV	
	Joint 1	Joint 2	Joint 1	Joint 2	Joint 1	Joint 2
**Case 1**	3.8499	2.8982	108.4324	102.3203	43.3315	48.9875
**Case 2**	4.8416	3.3159	156.3586	124.0548	45.3231	51.1957
**Case 3**	4.5843	3.1525	133.4075	114.3752	49.8185	53.9525
**AGITSMC**	3.6090	2.7152	96.6866	95.7613	41.5689	46.5755

## Data Availability

The original contributions presented in this study are included in the article. Further inquiries can be directed to the corresponding author.
